# Spatiotemporal 3D chromatin organization across multiple brain regions during human fetal development

**DOI:** 10.1038/s41421-025-00798-w

**Published:** 2025-05-16

**Authors:** Yaoyu Sun, Min Li, Chao Ning, Lei Gao, Zhenbo Liu, Suijuan Zhong, Junjie Lv, Yuwen Ke, Xinxin Wang, Qiang Ma, Zeyuan Liu, Shuaishuai Wu, Hao Yu, Fangqi Zhao, Jun Zhang, Qian Gong, Jiang Liu, Qian Wu, Xiaoqun Wang, Xuepeng Chen

**Affiliations:** 1 GMU-GIBH Joint School of Life Sciences, The Guangdong-Hong Kong-Macau Joint Laboratory for Cell Fate Regulation and Diseases, Guangzhou National Laboratory, Guangzhou Medical University, Guangdong, China; 2 Key Laboratory of Epigenetic Regulation and Intervention, Institute of Biophysics, Chinese Academy of Sciences, Beijing, China; 3 University of Chinese Academy of Sciences, Beijing, China; 4 State Key Laboratory of Brain and Cognitive Science, Institute of Biophysics, Chinese Academy of Science, Beijing, China; 5 National Laboratory of Biomacromolecules, Institute of Biophysics, Chinese Academy of Science, Beijing, China; 6 State Key Laboratory of Cognitive Neuroscience and Learning, Beijing Normal University, Beijing, China; 7 IDG/McGovern Institute for Brain Research, New Cornerstone Science Laboratory, Beijing Normal University, Beijing, China; 8 College of Biological Science, China Agricultural University, Beijing, China; 9 Changping Laboratory, Beijing, China; 10 Obstetrics and Gynecology Medical Center of Severe Cardiovascular of Beijing Anzhen Hospital, Capital Medical University, Beijing, China; 11 The Fifth Affiliated Hospital of Guangzhou Medical University, Guangzhou Medical University, Guangdong, China

**Keywords:** Chromatin structure, Epigenetics

## Abstract

Elucidating the regulatory mechanisms underlying the development of different brain regions in humans is essential for understanding advanced cognition and neuropsychiatric disorders. However, the spatiotemporal organization of three-dimensional (3D) chromatin structure and its regulatory functions across different brain regions remain poorly understood. Here, we generated an atlas of high-resolution 3D chromatin structure across six developing human brain regions, including the prefrontal cortex (PFC), primary visual cortex (V1), cerebellum (CB), subcortical corpus striatum (CS), thalamus (TL), and hippocampus (HP), spanning gestational weeks 11–26. We found that the spatial and temporal dynamics of 3D chromatin organization play a key role in regulating brain region development. We also identified H3K27ac-marked super-enhancers as key contributors to shaping brain region-specific 3D chromatin structures and gene expression patterns. Finally, we uncovered hundreds of neuropsychiatric GWAS SNP-linked genes, shedding light on critical molecules in various neuropsychiatric disorders. In summary, our findings provide important insights into the 3D chromatin regulatory mechanisms governing brain region-specific development and can serve as a valuable resource for advancing our understanding of neuropsychiatric disorders.

## Introduction

A human brain is a highly complex organ that consists of distinct anatomical and functional brain regions. The human brain performs high-level cognitive functions, sensory perception, memory, and movement control through these specialized anatomical brain regions. During the development of the brain, the anterior part of the neural tube expands and forms three primary brain vesicles — the prosencephalon (forebrain), mesencephalon (midbrain), and the rhombencephalon (hindbrain). Following subsequent divisions into the secondary brain vesicles, the forebrain generates the telencephalon and diencephalon, the midbrain gives rise to sub-compartments, and the hindbrain gives rise to the metencephalon and myelencephalon. Then, the developing neurons and glial cells migrate, differentiate, and form different specialized brain regions, including cerebral cortex, striatum, thalamus (TL), hippocampus (HP), and cerebellum (CB).

The regionalization and development of different brain regions in humans require fine-tuned gene regulation and spatial-temporal orchestration. Recent surveys have shed light on the transcriptome during the development of the human cerebrum and cerebellum^1–6^. The three-dimensional (3D) chromatin structure plays important roles in the mammalian nervous system development^7–14^. However, 3D chromatin structure landscapes during the development of different brain regions in humans remain poorly understood. 3D chromatin structure reorganization is tightly associated with gene expression regulation^15–17^, embryonic development^18–21^ and disease pathogenesis^22,23^. Physical chromatin loops can bring distal *cis* elements to the target gene promoter in a proximal 3D space to regulate gene expression^8,16,24–26^. It has been reported that the 3D chromatin structure dynamics can lead to gene expression alterations and eventually contribute to unique human brain phenotypes during primate corticogenesis^27^. Although some previous pioneering studies investigated chromatin contacts in the developing human cerebral cortex^7,10,28^, high-resolution 3D chromatin structure landscapes in other functional brain regions are still scarce, which limits the understanding of different brain region-specific characteristics in humans.

Here, we collected six different major brain regions at the human fetal mid-gestation stage and generated high-resolution 3D chromatin structure landscapes. By multi-omics integration in different developing brain regions, we identified many brain region-specific 3D chromatin structures. These region-specific 3D chromatin structures provide us with biological and functional insights for understanding regulatory mechanisms of the development of different brain regions in humans.

## Results

### Mapping 3D chromatin organizations in six developing human brain regions

**Fig. 1:**
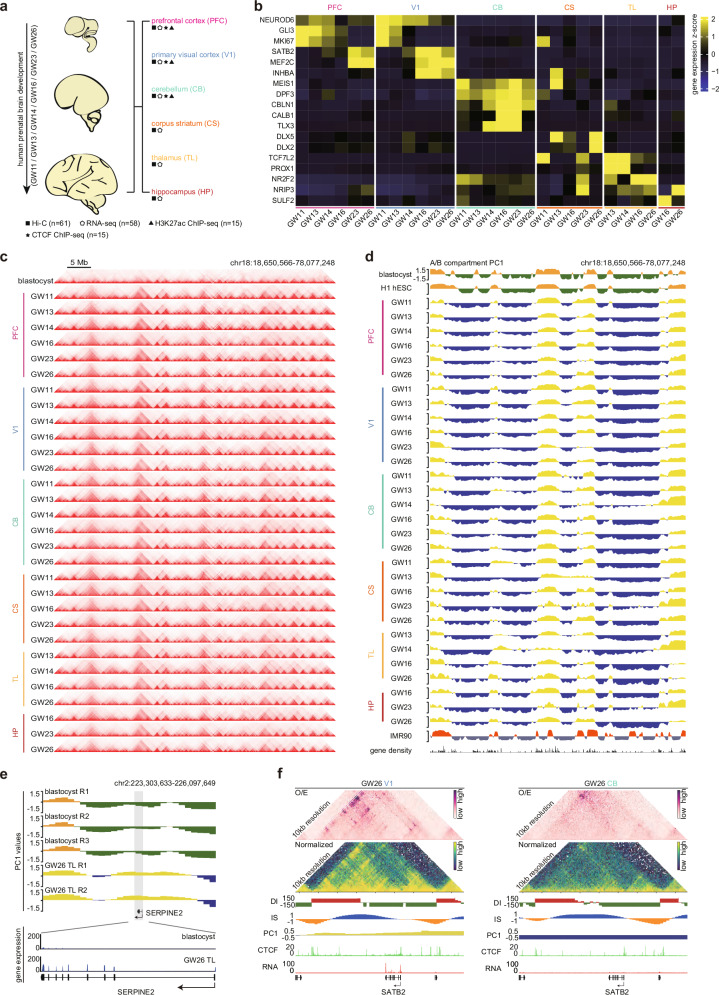
3D chromatin landscapes in multiple developing human brain regions. **a** The overall study design for spatiotemporal 3D chromatin organization of multiple developing brain regions in humans. **b** The gene expression heatmap for representative genes of different brain regions. **c** Heatmaps for TADs on chromosome 18 in the blastocyst and 30 developing human brain region samples. **d** A/B compartment PC1 tracks for the same genomic region as (**c**) in 30 developing human brain region samples and the blastocyst, H1 hESC, IMR90 cells, along with gene density track below. **e** The snapshot showing the A/B compartment switches and gene expression patterns for the SERPINE2 gene in the blastocyst and the GW26 TL sample. **f** The observed/expected (O/E) heatmaps and interaction heatmaps at 10-kb resolution for the SATB2 gene in V1 and CB brain regions at the GW26, along with Directional Index (DI) tracks, Insulation Score (IS) tracks, A/B compartment PC1 tracks, CTCF ChIP-seq tracks, and gene expression tracks below.

To map 3D chromatin organization landscapes and investigate their regulatory functions in different brain regions during fetal development in human, we collected six distinct developing brain regions according to their anatomical locations and anatomical characteristics. We collected cerebral prefrontal cortex (PFC), primary visual cortex (V1), cerebellum (CB), subcortical corpus striatum (CS), thalamus (TL), and hippocampus (HP) during the period from the gestational week (GW) 11–26 stage which mainly encompasses neurogenesis and synaptogenesis (Fig. 1a and Supplementary Fig. S1a–c, see the “Materials and methods” section). We performed deep in-situ Hi–C sequencing for 3D chromatin structures and RNA-seq for gene expression in these different developing brain samples. We also performed CTCF ChIP-seq to identify CTCF-binding landscapes and H3K27ac ChIP-seq to predict enhancers (Fig. 1a and Supplementary Fig. S1a–c).

As expected, the gene expression showed that different brain regions specifically expressed the corresponding brain region markers (Fig. 1b). For example, the human PFC and V1 specifically express cerebral cortical markers such as *SATB2* and *NEUROD6*, and the human TL highly express thalamic neurons marker gene *TCF7L2*^29^ (Fig. 1b). Next, our Hi–C data include more than 33 billion reads for 30 samples from 6 individuals. We got an average of 460 million valid reads for each Hi–C sample (Supplementary Fig. S1a and Table S1). The Hi–C data replicates in different brain regions show high reproducibility (Supplementary Fig. S1d–f). We could observe clear topologically associating domains (TADs) at 40-kb resolution and A/B compartments at 100-kb resolution in multiple developing human brain regions, which showed obvious differences from the human blastocyst and H1 ESCs (Fig. 1c, d). Taken together, we generated an atlas of high-resolution spatiotemporal 3D chromatin organization landscapes in six developing human brain regions.

### The dynamic A/B compartments in developing human brains

After the blastocyst stage, different tissue lineages begin to emerge and specialize. A/B compartments are closely associated with gene expression regulation^30,31^. At different mid-gestation stages, our data showed that the developing brain region samples were distinguished from the human blastocyst, H1/H9 stem cells, and IMR90 fetal lung cells (Fig. 1d and Supplementary Fig. S1e). According to the hierarchical clustering of A/B compartments, the PFC and V1 regions were similar at earlier stages (GW11/GW13/GW14) but gradually showed differences at later stages (GW23/GW26) (Supplementary Fig. S1e). Besides, the CS region was globally similar to the CB region at the GW11 stage and GW13 stage, but after the GW16 stage, the CS region became more similar to the PFC and V1 region (Supplementary Fig. S1e). These results indicated dynamic A/B compartments for different brain regions during a gradual regionalization in developing human brains.

Next, we wanted to know how the dynamics of A/B compartments influenced the functions of different brain regions in humans. We classified the A/B compartment dynamic regions from the human blastocyst to the different developing brain regions and performed GO biological process enrichment analysis for genes with A/B compartment switches in the six brain regions (Supplementary Fig. S2a and Table S2). There were almost 31% regions with dynamic A/B compartments in the developing human brain (Supplementary Fig. S2b). Generally, genes from the B to A compartment switched clusters were significantly upregulated than the expression of genome-wide genes, whereas genes from the A to B compartment switched clusters were significantly downregulated than genome-wide genes (Supplementary Fig. S2c). It was suggested that dynamic A/B compartments indeed changed gene expressions in developing human brains. For the function of genes with dynamic A/B compartments, we took the TL sample as an example and found that genes with A compartment status in the TL region but B compartment status in the human blastocyst, such as *SERPINE2* and *GRIA2*, showed the enrichment in hypothalamus development, glutamate receptor signaling pathway (Fig. 1e and Supplementary Fig. S2a, d). Similar analyses were also performed for the developing PFC, V1, CB, CS, and HP regions (Supplementary Fig. S2a). We found that the dynamic A/B compartment switches during brain development could be involved in the nervous development regulations (Supplementary Fig. S2a). For example, the results showed that genes from the glutamatergic synaptic transmission process would undergo the B-to-A compartment switches from the earlier GW11/GW13 CS to the later GW23/GW26 CSs (Supplementary Fig. S2a). Additionally, genes with A compartment status in the PFC region but B compartment status in the human blastocyst (cluster 1 and cluster 2) are significantly enriched in forebrain development, axon guidance, and regulation of synapse assembly (Supplementary Fig. S2a). A representative example is shown around the *MEF2C* gene, which played crucial roles for neuronal development^32,33^, with A compartment status and high gene expression in the later PFCs (GW23/GW26) but not in the human blastocyst and earlier PFCs (GW11/GW13/GW14) (Supplementary Fig. S2a, e). By contrast, genes with A compartment status in the human blastocyst but B compartment status in the PFC region (cluster 4) are enriched in blastocyst development and endocardium development (Supplementary Fig. S2a). At the genomic locus around the *OVOL2* gene that functions in human blastocyst development, we can observe A compartment status and high gene expression in the human blastocyst but not in the PFC region (Supplementary Fig. S2f).

Taken together, our results suggest that the dynamics of A/B compartments in developing human brains are tightly associated with the brain regional functions in the developing human brain.

### Divergent development of cortical subdivision PFC and V1 according to the 3D chromatin organization

**Fig. 2:**
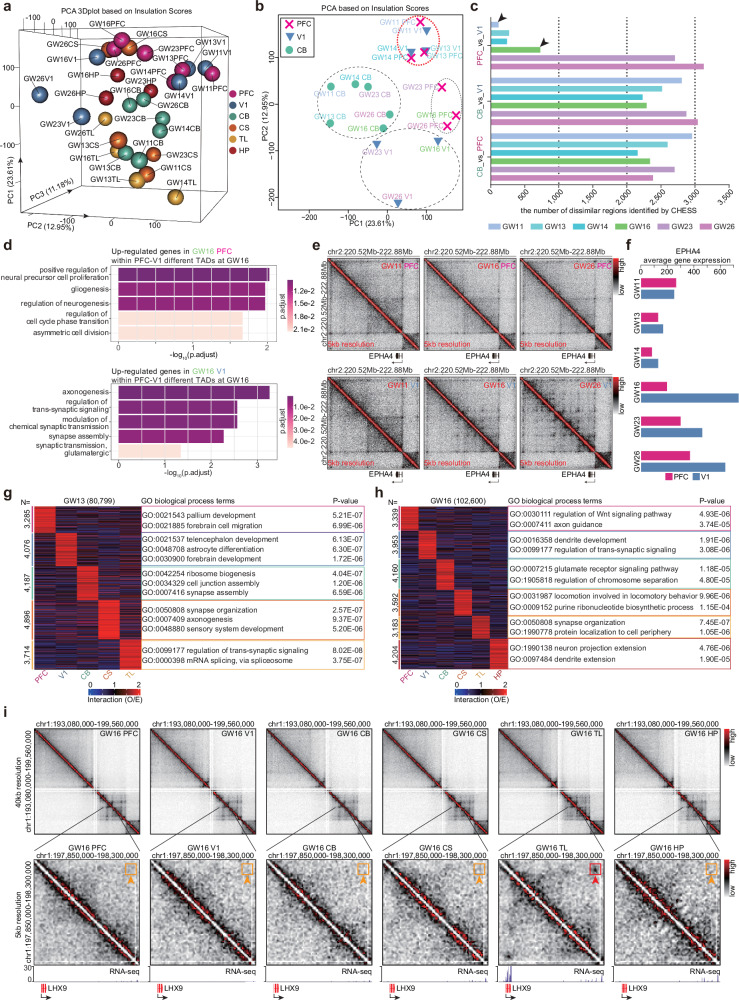
The dynamic 3D chromatin organization across multiple human brain regions. **a** The 3D plot of PCA analysis based on insulation scores for multiple developing human brain samples. The proportion of variances explained by the PC1, PC2, and PC3 values was also shown, respectively. **b** The 2D PCA plot for developing PFC, V1, and CB samples based on the insulation score. Proportions of variance explained by the PC1 and PC2 values were also shown. **c** Barplots for the number of dissimilar genomic regions between PFC, V1, and CB samples at each developmental stage. The dissimilar genomic regions were identified by CHESS software. **d** GO biological process enrichment barplots for genes within PFC-V1 different TADs at the GW16 stage. **e** Interaction heatmap examples at 5-kb resolution for the GW16 PFC-V1 different TADs around the *EPHA4* gene. **f** The *EPHA4* gene expression barplot of PFC and V1 samples from the GW11 to GW26 stage. **g**, **h** The O/E heatmaps for brain region-specific loops and their function enrichments at the GW13 stage (**g**) and at the GW16 stage (**h**). **i** An example for the TL region specific loops around the *LHX9* gene at the GW16 stage.

Insulation scores can measure the interaction insulation effect of one region across its neighboring loci and can reflect TAD domain structures^18,21,34^. We next investigated the dynamics of TAD domains across different brain regions (Fig. 1f). The PCA analysis based on insulation scores indicates that there are obvious 3D chromatin organization differences across different brain regions (Fig. 2a and Supplementary Fig. S3a). The PFC, V1, and HP samples tended to cluster together, whereas the CB and TL samples were separated from the PFC, V1, and HP samples (Fig. 2a).

Furthermore, although the PFC samples and the V1 samples both belong to the cerebral cortex, these two cortical subdivisions are responsible for distinct brain functions. The PFC region, the front part of the frontal brain lobe, is responsible for cognition, decision-making, emotion, and social behavior^35–38^. The V1 region, located around the calcarine fissure in the occipital lobe, is the area of the cerebral cortex that processes visual information^39^. We observed that the PFC and V1 samples at the GW11/GW13/GW14 stages tended to cluster closely (Fig. 2a, b). However, from the GW16 stage onwards, PFC and V1 samples showed obvious differences (Fig. 2a, b). By contrast, the CB samples were always separated from PFC and V1 samples (Fig. 2b). Furthermore, the observation above was also supported by the CHESS analysis, which was a feature-free method robustly comparing chromatin contact maps^40^. In line with the insulation score PCA result, there were limited differential 3D chromatin regions between PFC and V1 before the GW14 stage, the difference of 3D chromatin regions between PFC and V1 emerged at the GW16 stage and became more evident at GW23/GW26 stages according to the CHESS analysis (Fig. 2c and Supplementary Fig. S3b). By contrast, the 3D chromatin regions of the CB samples showed significant differences from the PFC and V1 samples even as early as the GW11 stage (Fig. 2c and Supplementary Fig. S3c). We then identified the PFC-V1 different TADs at the GW16 stage and investigated how it was related to the divergent development of these two cortical subdivisions. Our results showed that PFC upregulated genes within the PFC-V1 different domains were enriched in the positive regulation of neural precursor cell proliferation and asymmetric cell division, whereas V1 upregulated genes were enriched in the regulation of trans-synaptic signaling and synapse assembly (Fig. 2d). For example, the domain containing V1-highly-expressed gene *EPHA4* had no significant differences between PFC and V1 samples at the GW11 stage, but the differences arose at the GW16 stage accompanied with the significant gene expression up-regulation in the V1 compared to the PFC (Fig. 2e, f). Additionally, we identified genomic regions with differential insulation scores in the PFC and V1 from GW23/GW26 stages and profiled CTCF-binding signals along these regions. We could clearly observe the CTCF-binding enrichment along the domain boundaries as expected, but no enrichment for the differentially insulated regions (Supplementary Fig. S3d).

The PFC samples and the V1 samples also had very limited region-specific genes at the GW11 stage (Supplementary Fig. S4a). At the GW16 stage, region-specific genes become more abundant between the PFC region and the V1 region (Supplementary Fig. S4a). PFC-specific genes were usually enriched in extracellular matrix organization and regulation of neural precursor cell proliferation, whereas V1-specific genes were enriched in regulation of trans-synaptic signaling and synapse organization at the GW16 stage (Supplementary Fig. S4b, c).

Taken together, our results suggested that although both the PFC region and the V1 region, belong to the cerebral cortex, 3D chromatin domain reorganizations still occurred between these two cortical subdivisions at the GW16 stage, which contributed to the divergent development of these two subdivisions.

### Brain region-specific and developmental stage-specific chromatin loops in developing human brains

Additionally, communications via loops between genes and *cis*-regulatory elements are very important for gene expression regulation, cell identity, and development^16,24,28^. To investigate the regulatory roles of dynamic loops in different brain regions, we totally identified more than 428,000 loops in all six developing brain regions (Supplementary Fig. S5a and Table S3). Expectedly, most of the loops were also located within TADs in developing brain regions (Supplementary Fig. S5b).

We next identified region-specific loops across multiple brain regions at each stage (Fig. 2g, h and Supplementary Fig. S5c–f). The interaction observed/expected (O/E) values of chromatin loops supported the region specificity of developing human brains (Fig. 2g, h and Supplementary Fig. S5c–f). We performed the GO enrichment analysis for genes whose promoters were linked with region-specific chromatin loops. Our results showed that the region-specific loop-linked genes are significantly enriched in brain region-associated neural development processes (Fig. 2g, h and Supplementary Fig. S5c–f). Genes with the PFC-specific loops at the GW13 stage showed a significant enrichment in pallium development and forebrain cell migration (Fig. 2g). At the GW16 stage, the V1-specific loop-linked genes were enriched in dendrite development and regulation of trans-synaptic signaling, and TL-specific loop-linked genes were enriched in synapse organization and protein localization to cell periphery (Fig. 2h). We observed a TL-specific loop for the *LHX9* gene that was a transcription factor controlling thalamic neuron development^41^, and *LHX9* gene was highly expressed in the TL region (Fig. 2i). In addition, we also found many telencephalon-specific loops around relevant cortical neuronal genes, such as *FOXG1* (Supplementary Fig. S5g). The *FOXG1* gene functions in the establishment of the cortical subdivision of the developing brain^42^. We can observe strong loops for *FOXG1* gene promoters in the PFC, V1, and CS regions and weak loops in the HP region, but these loops disappeared in the CB and TL regions at the GW16 stage (Supplementary Fig. S5g).

Besides brain region-specific chromatin loops, we also found thousands of developmental stage-specific chromatin loops in the developing human brain (Supplementary Fig. S6a–f). Our results showed that genes with the early stage (GW11/GW13)-specific loops were usually enriched in regulation of mitotic cell cycle, mRNA splicing and epithelial cell proliferation, whereas genes with the late stage (GW23/GW26)-specific loops were enriched in negative regulation of canonical Wnt signaling pathway and branching morphogenesis of a nerve (Supplementary Fig. S6a–f). We also identified thousands of gene-loop pairs showing strong correlations between loop strength and gene expressions, such as TCF7L2 (Supplementary Fig. S6g).

Collectively, our results suggested that there were extensive brain region-specific and developmental stage-specific chromatin loops in the developing human brain, which widely participated in the functions of different brain regions.

### Super-enhancers (SEs) in the human developing brain

SEs were reported to be associated with gene regulation and can strongly contribute to the cell/tissue fate identity^43^. Therefore, we next explored H3K27ac-marked SEs in the developing human brain and their associated loops. We first predicted hundreds of SEs in multiple human brain regions (Supplementary Fig. S7a, b). For example, around the *MIR9-3HG* locus, we could find SEs in the PFC, V1, and CB samples with strong H3K27ac signal, but this region was not an SE in H1 ESCs and the IMR90 cells (Supplementary Fig. S7c). We could observe strong enrichment of H3K27ac signal in SEs of the human developing brains, but not in the randomly shuffled control regions (Supplementary Fig. S7b). Furthermore, we also identified many brain region-specific SEs, such as a PFC/V1-specific SE in the *PDE2A* gene locus (Supplementary Fig. S7d).

**Fig. 3:**
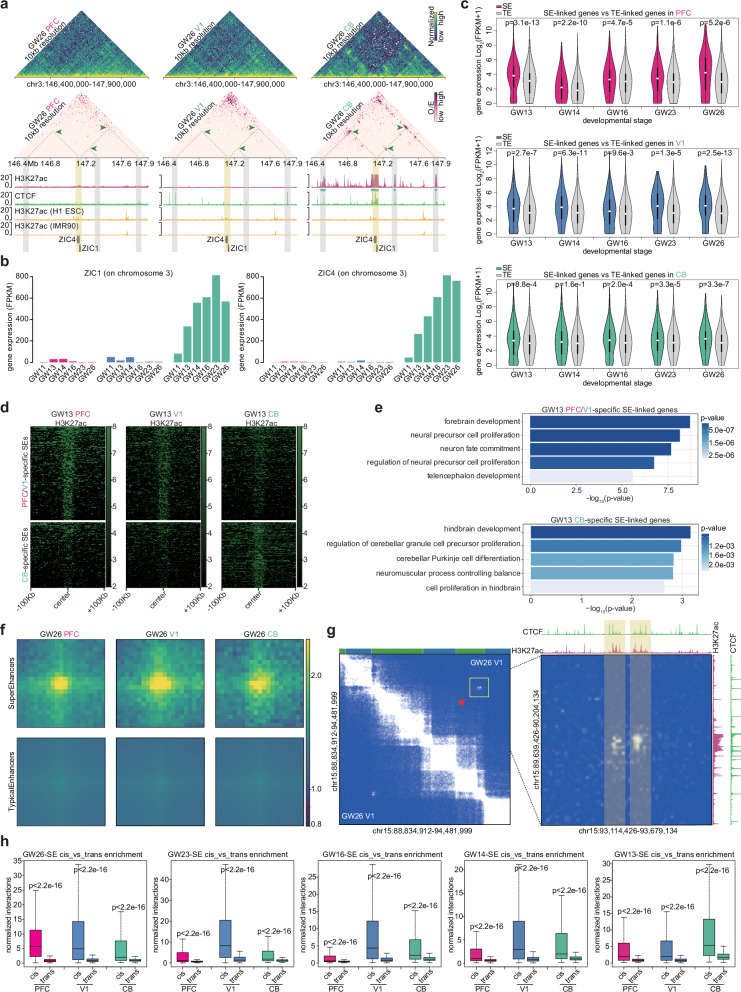
SEs and SE-associated loops in the human developing brain. **a** Ten-kb resolution O/E interaction heatmaps around *ZIC1/ZIC4* gene overlaid with brain H3K27ac ChIP-seq tracks, brain CTCF ChIP-seq and H1/IMR90 H3K27ac ChIP-seq tracks. Green arrows indicated dynamic loops mediated by H3K27ac-marked SEs in the CB region. **b** The *ZIC1/ZIC4* gene expression barplot of PFC, V1 and CB samples from the GW11 to GW26 stage. **c** The violin plots for SE-linked genes and TE-linked genes in the developing human brain region. **d** H3K27ac signal enrichment heatmaps around the differential SEs between the developing PFC/V1 region and the CB region at the GW13 stage. **e** Barplots of GO biological process enrichment for region-specific SE-linked genes at the GW13 stage. The top panel is for GW13 PFC/V1-specific SE-linked genes, and the bottom panel is for GW13 CB-specific SE-linked genes. **f** The respective enrichment plot for interactions between identified SEs and interactions between TEs in the GW26 PFC, the GW26 V1, and the GW26 CB. **g** An example for TAD crossing interactions between SEs. **h** Boxplots of the interaction enrichment for cis SE − SE interactions and trans SE–SE interactions from the GW13 to the GW26 stage. One-sided Wilcox. test for calculating the statistical significance.

Among brain region-specific loops in the human developing brain, we observed that there were many loops mediated by H3K27ac-marked SEs in a brain region-specific manner. We found 89 genes with significant association for SE-linked loops and expression (Supplementary Fig. S8a). For example, at the *ZIC1/ZIC4* gene locus, we could observe that CB-specific SEs had chromatin loops with *ZIC1/ZIC4* genes, but these loops and SEs disappeared in the PFC and V1 samples (Fig. 3a). *ZIC1* and *ZIC4* genes were also highly expressed in the CB samples (Fig. 3b). *POU3F3* gene played important roles in the cortical development^44^. We could also observe that at the *POU3F3* locus, there were PFC/V1-specific loops connecting the *POU3F3* gene and the PFC/V1-specific SEs, but it did not occur in the CB region (Supplementary Fig. S8b). We further found that the expression levels for the SE-linked genes were significantly higher than that for the typical enhancer (TE) linked genes in the developing human brain (Fig. 3c). We performed biological process enrichment for brain region-specific SE linked genes, and found that they were significantly related to region-specific brain functions (Fig. 3d, e). For example, the CB region-specific SE-linked genes were enriched in the hindbrain development and cerebellar Purkinje cell differentiation (Fig. 3e).

In addition, we strikingly found that compared to TEs, there were strong interactions between SEs, suggesting that SEs prefer to contact each other and form significant interactions (Fig. 3f and Supplementary Fig. S8c). For example, we could observe two very long-range significant interactions across several TADs in the V1 region for the two SEs (Fig. 3g). Furthermore, we found that the SE-SE contacts usually occurred within the same chromosome rather than across different chromosomes, showing a cis interaction enrichment (Fig. 3h).

Taken together, we found hundreds of SE, and they could mediate very long-range interactions crossing TADs in multiple human brain regions. Besides it, brain region-specific SEs and their associated loops could regulate region-specific gene expressions.

### Knockout of SLN’s enhancer could reduce the neuron maturation

**Fig. 4:**
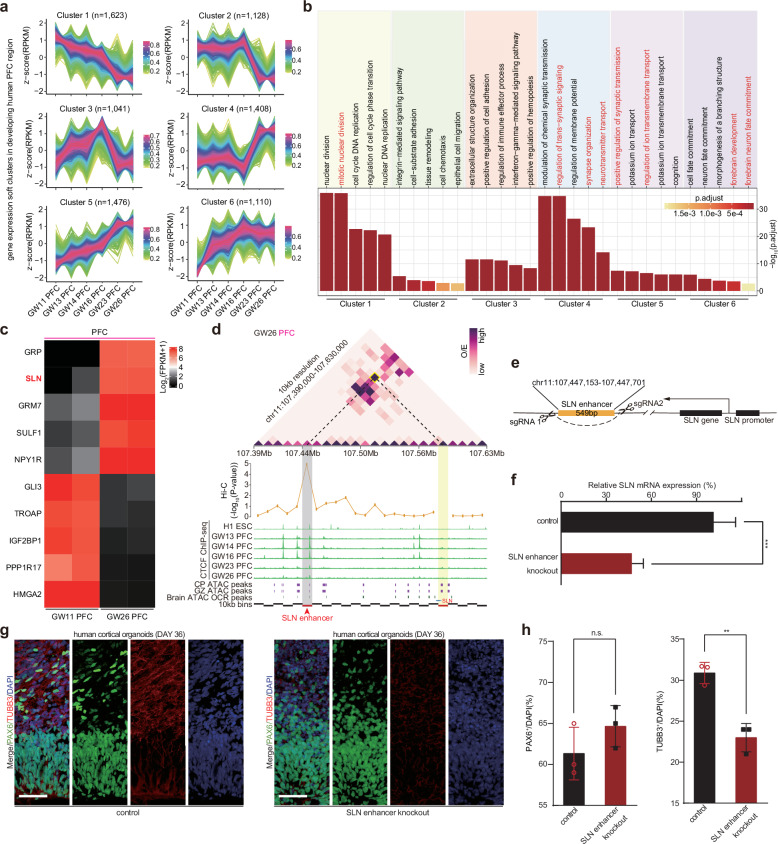
The knockout of SLN’s enhancer can reduce the cortical neuron maturation. **a** The dynamic soft clustering of genes during the development of the human PFC region. **b** The GO biological process enrichment results for dynamic gene clusters of the human PFC region. **c** The gene expression heatmap for top differential genes between GW11 PFC and GW26 PFC. **d** The 10-kb resolution O/E heatmap and interaction tracks for *SLN* gene promoter along with H1 and PFC CTCF ChIP-seq and published CP/GZ ATAC peaks (GSE95023), and brain ATAC OCR regions (GSE149268). **e** The schematic cartoon for *SLN* enhancer knockout. Predicted cleavage sites for two guide RNA pairs are shown. **f** The gene expression level for control (*n* = 3) and *SLN* enhancer knockout samples (*n* = 3). Error bars represent mean ± SEM. ***P < 0.001 (two-sided *t*-test). **g** Immunostaining for PAX6/TUBB3/DAPI at the human cortical organoid development day 36. Scale bars, 50 μm. Representative images are shown from *n* = 3 indepe*n*dent replicates. **h** Barplots for the percentage of PAX6^+^ neuron progenitor cells and the percentage for TUBB3^+^ mature neuron cells in control (*n* = 3) and *SLN* enhancer-knockout samples (*n* = 3). Error bars represent mean ± SEM. n.s., no significance (two-sided *t*-test).

To investigate the regulatory functions of 3D chromatin organizations during brain development, we calculated gene clusters according to the gene expression dynamic patterns, which could provide us with developmental details for different brain regions in humans (Fig. 4a, b and Supplementary Fig. S9a-d). For example, the TL is a central relay station between the sense organs and higher brain regions, playing roles in regulating sleep/wake rhythms^45^. We could observe that in the TL region, the cluster 6 genes whose gene expressions persistently increased during the TL development were responsible for the regulation of circadian sleep/wake cycle (Supplementary Fig. S9d).

In the PFC region, the GW23/GW26 PFCs tended to express genes responsible for positive regulation of synaptic transmission and regulation of ion transmembrane transport (Fig. 4a, b). *SLN* (*Sarcolipin*) gene was among the top upregulated genes from the GW11 to the GW26, and was preferentially expressed in GABAergic neurons (Fig. 4c and Supplementary Fig. S10a–c). For a longer developmental time window from the BrainSpan database^46^, we could also find that the SLN gene mainly expresses in the late-mid-prenatal stage and the late-prenatal stage, in accordance with the SLN gene expression dynamics in our data (Supplementary Fig. S10b). Therefore, we next focused on the SLN gene and wanted to study its regulation according to the 3D chromatin organization in the PFC.

According to the O/E heatmap and virtual 4 C chromatin interaction track, we could identify a new regulatory enhancer for the *SLN* gene (Fig. 4d and Supplementary Fig. S10d). A genomic bin locating at 140 kb downstream of *SLN* gene had a significant interaction with the *SLN* promoter in the GW26 PFC region (Fig. 4d). Within the dynamic chromatin interaction anchor, we find a CTCF-bing site overlapped with open chromatin peaks^7,47^ as the *SLN’s* enhancer (Fig. 4d). Then, we used CRISPR/Cas9 to successfully eliminate the SLN enhancer site in human H9 embryonic stem cells (Fig. 4e, f, Supplementary Fig. S10e, and Supplementary Data Source 1). We continued to culture the embryonic stem cells into the *SLN*-enhancer-knockout human cortical organoids and the control human cortical organoids. We further examined the neuron cell number in the organoid by immunostaining for neuron progenitor cell maker PAX6 and mature neuron marker TUBB3 (Fig. 4g). Intriguingly, the cell number was significantly reduced for mature neurons but not for progenitor cells in the *SLN*-enhancer-knockout organoids when compared to the control, indicating that the *SLN* enhancer could play roles in neuron maturation (Fig. 4h). Additionally, the size of the *SLN-*enhancer-knockout human cortical organoids were also significantly smaller than the control (Supplementary Fig. S10f, g).

Collectively, our results showed that the knockout of SLN’s enhancer could reduce the neuron maturation. We took a late-stage PFC upregulated gene, SLN, as an example and validated that regulatory enhancers predicted by 3D chromatin interaction data in this study could indeed influence neuron development.

### The investigation of virtual cell types in developing human brains

There are many different kinds of neurons and non-neurons in the human brain. It is worth unveiling which cell types exist in the developing human brain and their proportions for better understanding the 3D genome regulation. There were previously published single-cell RNA-sequencing (scRNA-seq) data for the developing human brains^3,4,6^, which generate delicate findings on complex brain cell anatomies. Next, we used the published scRNA-seq data^6^ to deconvolute our bulk RNA-seq data to gain a basic knowledge of the virtual cell types in our samples. With the deconvoluted transcriptomic results, we can see that the NPC-Gluta neuron transition occurred from the GW11 PFC to GW26 PFC (Supplementary Fig. S11a). In the GW16 PFC, there was a greater proportion of NPCs than that in the GW16 V1, which showed a similar result with the observation that GW16 V1 expressed more mature neuronal genes than GW16 PFC in Supplementary Fig. S4c. As expected, we observed that Cerebellar granule cells (Cere_GC) and Purkinje cells composed the majority of the CB samples from the GW13 to the GW23 stages (Supplementary Fig. S11a). The CS samples are complex with different proportions of NPCs, GABA_Neurons, Gluta_Neurons, and Astrocytes (Supplementary Fig. S11a).

Then, we wanted to investigate the virtual cell types for our Hi–C data. We collected the single-cell multi-omic Hi–C data from the adult human brain^11^. It can, to some degree, help us to know possible virtual cell types in our developing human brain Hi–C samples. We calculated the correlation of our Hi–C datasets from each GW stage to the published adult single-cell Hi–C data. The result showed that our Hi–C datasets from the developing human brain had a higher correlation with excitatory and inhibitory neurons than other non-neuronal cells, suggesting that our Hi–C data were largely contributed by neurons (Supplementary Fig. S11b).

More importantly, these adult brain samples covered the cerebral cortex, V1, and basal nuclei, which provide possible comparisons between developing brains and adult brains. First, we mapped the adult brain interactions on the developing brain domains. The result showed that the adult brain has obvious enrichment on the developing brain domains, suggesting that they probably shared a similar global domain segmentation (Supplementary Fig. S11c). Furthermore, we compared the AB-compartment strength between developing brains and adult brains. We could find that there was an increased AB-compartment strength from the developing brains to adult brains (Supplementary Fig. S11d). It was accompanied by the loss of A−B interactions crossing different compartment states, which can be exemplified by the loss of inter-compartment interactions in the GW26 CS and CaB (adult) on chromosome 3 (Supplementary Fig. S11e).

Together with the published single-cell RNA and Hi–C data, we investigated the virtual cell types within developing human brains used for this study. These virtual cell type compositions provide us with details to better interpret our chromatin structure data, and inter-compartment interactions would decrease during brain development into the adult, especially for the corpus striatum.

### The SNP-linked genes for neuropsychiatric disorder/trait GWAS variants

**Fig. 5:**
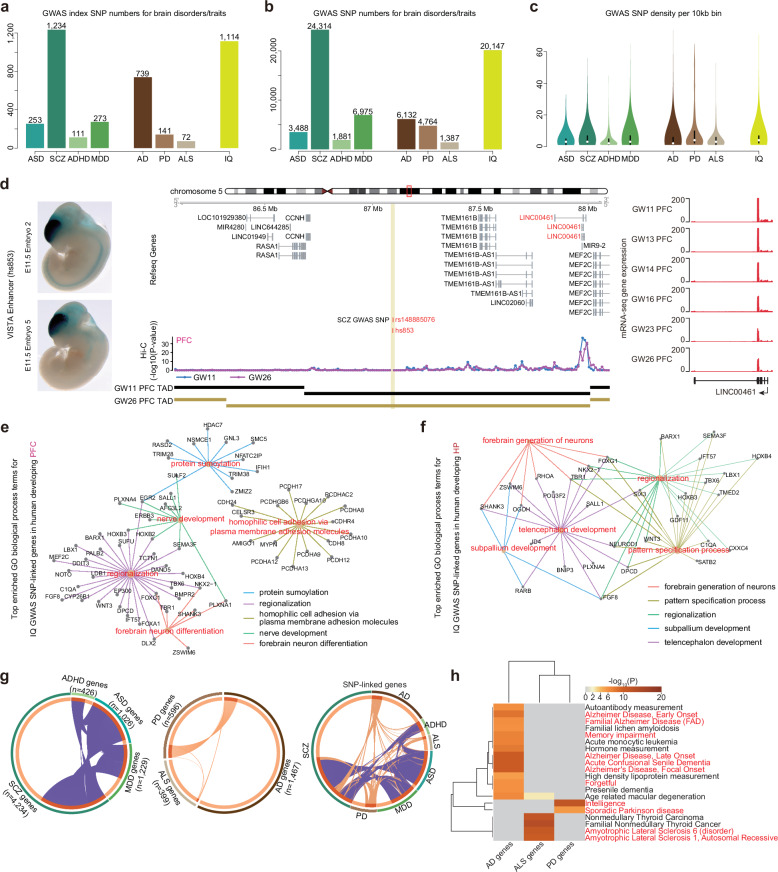
The SNP-linked genes for neuropsychiatric disorder/trait GWAS SNPs. **a** The GWAS index SNP summary for 8 neuropsychiatric disorders/traits. **b** The total GWAS LD-restricted SNPs for 8 neuropsychiatric disorders/traits. **c** The GWAS SNP density of 10-kb bins for 8 neuropsychiatric disorders/traits. **d** The interaction tracks of a SCZ-associated SNP *rs148885076* within a VISTA enhancer *hs853*. SCZ GWAS SNP *rs148885076* and its linked gene *LINC00461* were marked in red. LacZ staining results in mouse E11.5 embryos showing tissue-specific patterns of *hs853* enhancer activity (from VISTA enhancer browser). **e** The top enriched GO networks of IQ GWAS SNP-linked genes identified from the PFC region. **f** The top enriched GO networks of IQ GWAS SNP-linked genes identified from the HP region. **g** The circular overlap plot for genes of seven neuropsychiatric disorders (the left), genes across four neurodevelopmental disorders (ASD, ADHD, SCZ, and MDD) (the middle), and genes across three degenerative neurological diseases (AD, PD, and ALS) (the right). **h** The hierarchical clustering heatmap for disease catalog (from the DisGeNET) enrichment results of AD/PD/ALS GWAS SNP-linked genes.

Large-scale GWAS studies provide us with rich resources for understanding neuropsychiatric disorders/traits^48–50^. Given that most of GWAS SNPs were located in the non-coding intergenic regions, deciphering these SNP-associated genes in different brain regions can significantly enhance our understanding of neuropsychiatric disorders/traits. Hence, we next integrated 3D chromatin structures in different brain regions to interpret these neuropsychiatric disorder/trait GWAS SNPs. We summarized GWAS SNPs for several widely concerned neuropsychiatric disorders including autism spectrum disorder (ASD), attention deficit hyperactivity disorder (ADHD), schizophrenia (SCZ), major depressive disorder (MDD), Alzheimer’s disease (AD), Parkinson’s disease (PD), amyotrophic lateral sclerosis (ALS) and the trait of intelligence (IQ) based on the GWAS catalog (Fig. 5a–c, Supplementary Fig. S12a, b, and Table S4, see the “Materials and methods” section). The GWAS SNP number ranged from 1387 to 24,314 for these neuropsychiatric disorders/traits (Fig. 5b). Next, we assigned SNP-linked genes for these GWAS SNPs if the GWAS SNP had significant interactions with gene promoters at 10-kb resolution (see the “Materials and methods” section). With the chromatin interaction analysis, we systematically found hundreds of SNP-linked genes for these neuropsychiatric disorder/trait GWAS SNPs in six brain regions (Supplementary Fig. S12c). For example, the *rs148885076* SNP is a SCZ GWAS SNP that was located within a VISTA^51^ enhancer *hs853* showing an enhancer activity in mouse developing brains (Fig. 5d). We identified the *LINC00461* as *rs148885076* SNP-linked gene in the PFC region by chromatin interactions (Fig. 5d). This *LINC00461* gene is an evolutionarily conserved non-coding gene predominantly expressed in the brain, and reported to involve in gliomagenesis^52^. ADHD is a highly heritable childhood behavioral disorder. 5% of children and 2.5% of adults are suffering from ADHD^53^. The *rs16998572* SNP was significantly associated with ADHD according to the GWAS catalog. The chromatin interaction analysis of the *rs16998572* SNP showed that this SNP did not link the closest gene *BCAS1* but linked the *ZNF217* gene in the PFC region (Supplementary Fig. S12d). The *ZNF217* gene was highly expressed in the earlier PFC stages (Supplementary Fig. S12d), which was consistent with the role that *ZNF217* promoted cell proliferation and antagonized cell death^54^. In addition, we also identified SNP-linked genes for the IQ. The GO enrichment analysis showed that the IQ SNP-linked genes in the HP were significantly enriched in subpallium development and telencephalon development, and IQ SNP-linked genes in the PFC, such as *PCDHA8 and PLXNA1* genes, were significantly enriched in homophilic cell adhesion via plasma membrane adhesion molecules and forebrain neuron differentiation (Fig. 5e, f). Together, it was suggested that SNP-linked genes identified by chromatin interactions in multiple brain regions were significantly involved in the regulation of nervous development, underlying the molecular pathogenicity of neuropsychiatric disorders.

ASD, ADHD, SCZ, and MDD were generally considered as neurodevelopmental disorders, whereas AD, PD, and ALS were considered as neurodegenerative disorders. At last, we wanted to know whether there were some intrinsic connections for the neuropsychiatric disorders. We merged the SNP-linked genes of different brain regions together for each neuropsychiatric disorder and calculated the overlap of SNP-linked genes for these neuropsychiatric disorders. Our results showed that a large proportion of SNP-linked genes were shared by the neurodevelopmental disorders (SCZ, ASD, ADHD, and MDD), which was probably due to overlapping genetic basis of these diseases (Fig. 5g). On the contrary, only a few genes were shared for the neurodegenerative disorders (AD, PD, and ALS), suggesting distinct molecular mechanisms for different neurodegenerative disorders (Fig. 5g). We further performed the DisGeNET disease catalog enrichment and GO biological process enrichment for SNP-linked genes of AD, PD and ALS diseases (Fig. 5h and Supplementary Fig. S12e, f). The results supported that the neurodegenerative SNP-linked genes were specially enriched in their own typical disease phenotypes, such as AD SNP-linked genes enriched in the Memory impairment and Acute Confusional Senile Dementia phenotypes (Fig. 5h). The GO enrichment results showed that AD SNP-linked genes were enriched in the regulation of lipid localization and histone modifications, whereas PD SNP-linked genes were enriched in the steroid biosynthetic process and negative regulation of neuron projection development (Supplementary Fig. S12e, f). It was also indicated that different neurodegenerative disorders had their independent pathogenic molecules.

Collectively, our results suggested that 3D chromatin structures in multiple brain regions could help to define SNP-linked genes, especially for the intergenic GWAS SNPs, which laid a valuable foundation for interpreting the underlying functions of neuropsychiatric GWAS SNPs and understanding the molecular pathogenicity of neuropsychiatric disorders.

## Discussion

In this study, we generated an atlas of high-resolution 3D chromatin structure landscapes in multiple brain regions during the human mid-gestation development. The 3D chromatin structures showed obvious brain region-specific organization, including at the A/B compartment level, at the domain level, and at the chromatin loop level, which was tightly associated with the regulation of brain region-specific gene expressions and the development of brain regions. In the developing human brain, different brain regions need to take their own rhythm and orchestrate together through the fine-tuning of human brain development. We observed that PFC and V1 regions shared similar gene expression patterns at the GW11 stage but launched their own developmental programs and 3D chromatin structure organizations at the GW16 stage. However, questions about how cells in PFC and V1 regions communicate to synchronize their developmental paces at the early developmental stage and whether there existed region-specific neurotransmitters initiating their own developmental programs needed to be further studied. Additionally, 3D chromatin interactions in different brain regions could be used to interpret SNP-linked genes for neuropsychiatric disorder risk SNPs, which overcame the barrier of only interpretation in the cerebral cortex or in vitro neuronal cell lines.

Nevertheless, our study also suffered some limitations. The main weakness of this study is the lack of cell type specificity due to bulk brain samples. Recently, some advanced multi-omic technologies can capture chromosome structures and gene expressions simultaneously at single-cell levels, such as HiRES^55^, LiMCA^56^, scCARE-seq^57^, etc. Although we used the published scRNA-seq and single-cell adult brain Hi–C to improve the understanding of the cell type information in our bulk developing brain data, it is still expected to further study developing human brain chromatin structures and gene regulation with the advanced single-cell multi-omic technologies in the future.

In summary, this study revealed the spatiotemporal 3D chromatin organizations of multiple brain regions during fetal development in humans, allowing us to deeply decipher regulatory mechanisms of brain region-specific development.

## Materials and methods

No statistical methods were used to predetermine sample size. The experiments were not randomized, and investigators were not blinded to allocation during experiments and outcome assessment.

### Human ethics statement

The regulatory framework about the use of human fetal brain samples for this research was based on the policies of the Human Biomedical Research Ethics Guidelines (set by National Health Commission of the People’s Republic of China on Dec. 1st, 2016), the 2016 Guidelines for Stem Cell Research and Clinical Translation (issued by the International Society for Stem Cell Research, ISSCR) and the Human Embryonic Stem Cell Research Ethics Guidelines (set by China National Center for Biotechnology Development on Dec. 24, 2003). All the protocols are in compliance with the “Interim Measures for the Administration of Human Genetic Resources” administered by the Ministry of Science and Technology of China.

The human tissue collection and research protocols were approved by the Reproductive Study Ethics Committee of Beijing Anzhen Hospital (2014012x) and the Institutional Review Board of the Institute of Biophysics (H-W-20131104). All fetal brain samples were collected under standard clinical protocols after the donor patients signed an informed consent document.

### Fetal brain sample collection and dissection

De-identified fetal brain tissues with no karyotype abnormalities or genetic conditions reported were collected in fresh ice-cold artificial cerebrospinal fluid containing 125.0 mM NaCl, 26.0 mM NaHCO_3_, 2.5 mM KCl, 2.0 mM CaCl_2_, 1.0 mM MgCl_2_, and 1.25 mM NaH_2_PO_4_ at a pH of 7.4 when oxygenated (95% O_2_ and 5% CO_2_).

For different fetal brain regions, microdissections were performed carefully to sample target regions, including PFC, V1, CB, CS, TL, and HP. The collected fetal brain tissue was dissected and placed in Hibernate E medium (Invitrogen, A1247601), and then different brain region samples were stored in liquid nitrogen for subsequent experiments.

### In situ Hi–C library generation for human fetal brain samples

In situ Hi–C libraries for samples from different human fetal brain regions were performed according to previous protocols^16^. Concretely, the brain sample was fixed with 1 mL of freshly made 1% formaldehyde solution and incubated at room temperature for 10 min. To quench the reaction, 2.5 M glycine solution was added to a final concentration of 0.2 M. Samples were incubated at room temperature for 5 min and then centrifuged for 5 min at 3000× *g* at 4 °C. Discard supernatant. The pellet was washed with ice-cold 1× PBS and spun for 5 min at 3000× *g* at 4 °C. Discard supernatant and flash-freeze cell pellets in liquid nitrogen. Either proceed to the rest of the protocol or store cell pellets at −80 °C. The sample pellet was resuspended with 1 mL of ice-cold Hi–C lysis buffer (10 mM Tris-HCl, pH 8.0, 10 mM NaCl, 0.2% Igepal CA630) with a protease inhibitor cocktail. The cells incubate on ice for 30 min. Samples were then centrifuged at 3000× *g* for 5 min and the supernatants carefully discarded. Pelleted nuclei were washed once with 1 mL of ice-cold Hi–C lysis buffer. Discard the supernatant and resuspend the pellet with 50 μL of 0.5% sodium dodecyl sulfate (SDS). And incubated at 62 °C for 10 min. After incubating, 145 μL of water and 25 μL of 10% Triton X-100 were added to quench the SDS. Tubes were gently tapped to mix well, avoiding excessive foaming, and then incubated at 37 °C for 15 min. Twenty-five microlitres of 10× NEBuffer 2 U and 100 U of MboI restriction enzyme (NEB, R0147) were added, and chromatin was digested at 37 °C overnight with rotation. Samples were incubated at 62 °C for 20 min to inactivate MboI and then cooled to room temperature. To fill in the restriction fragment overhangs and mark the DNA ends with biotin, 50 μL of fill-in master mix (37.5 μL of 0.4 mM biotin-14-dATP, 4.5 μL of 10 mM dCTP/dGTP/dTTP mix, 8 μL of 5 U/μL DNA polymerase I, large) was added. Samples were mixed by pipetting and incubated at 37 °C for 1.5 h. Ligation master mix (663 μL of water, 120 μL of 10X NEB T4 DNA ligase buffer, 100 μL of 10% Triton X-100, 12 μL of 10 mg/mL bovine serum albumin, 10 μL of 400 U/μL T4 DNA ligase) was added, and samples were incubated at 16 °C for over 10 h with rotation. Nuclei were pelleted by centrifugation for 5 min at 3000× *g* and were washed with 1× PBS. Pellets were then resuspended in 400 μL 1× PBS, 16.7 μL 20 mg/mL proteinase K, and 40 μL 10% SDS, incubated at 55 °C for 30 min. The sample was added with 43.3 μL 5 M NaCl, 12 μL 0.5 M EDTA, 24 μL 1 M DTT, 50 μL 10% SDS, and incubated at 68 °C overnight. Cool the sample at room temperature, and add 1250 μL pure ethanol and 50 μL 3 M sodium acetate, pH 5.2. Mix by inverting and incubate at −80 °C for over 1 h. Centrifuge at 13,000× *g*, 4 °C for 20 min. Keep the tubes on ice after spinning and carefully remove the supernatant by pipetting. Wash with 800 μL of 70% ethanol twice and centrifuge at 13,000× *g* for 5 min. Dissolve the pellet in 100 μL of 1× Tris buffer (10 mM Tris-HCl, pH 8) and incubate at 37 °C for 15 min to fully dissolve the DNA. Quantify DNA by Qubit dsDNA High Sensitivity Assay (Life Technologies, Q32854). To make the biotinylated DNA suitable for high-throughput sequencing using Illumina sequencers, shear to a size of 300 − 500 bp. Transfer sheared DNA to a fresh 1.5 mL tube, and add an equal volume of 2× Binding Buffer (2× BB: 10 mM Tris-HCl, pH 7.5; 1 mM EDTA; 2 M NaCl), then centrifuge at 13,000× *g* for 5 min. Prepare for biotin pull-down by washing 50 μL of 10 mg/mL Dynabeads MyOne Streptavidin C1 beads (Life Technologies, 65002) with 400 μL of 1× Tween Washing Buffer (1× TWB: 5 mM Tris-HCl, pH 7.5; 0.5 mM EDTA; 1 M NaCl; 0.05% Tween 20). Separate on a magnet and discard the solution. Resuspend the beads with sheared DNA supernatant. Incubate at room temperature for 30 min with rotation to bind biotinylated DNA to the streptavidin beads. Separate on a magnet and discard the solution. Wash the beads by adding 500 μL of 1× TWB. Heat the tubes on a Thermomixer at 55 °C for 2 min with mixing. Reclaim the beads using a magnet and discard supernatant. Repeat wash twice. Resuspend beads in 100 μL 1× Tris buffer (10 mM Tris-HCl, pH 8) and transfer the beads to a new PCR tube. Reclaim beads and discard the buffer. Resuspend beads in 50 μL 1× Tris buffer (10 mM Tris-HCl, pH 8). Then the beads binding with fragmented DNA were treated with the End Repair/dA-Tailing Module (NEB, E7546L) and Ligation Module (NEB, E7595L) following the operation manual. After ligation, the beads were washed with 1× TWB twice and 1× Tris buffer (10 mM Tris-HCl, pH 8). Beads were resuspended in 20 μL of 1× Tris buffer (10 mM Tris-HCl, pH 8). The Hi–C library was amplified for 10 cycles of PCR with Q5 master mix (NEB, M0492L) following the operation manual. PCR products were confirmed by analyzing 1 μL of product using the FlashGel System (Lonza, 57063). PCR was continued with additional cycles until bright DNA bands were seen. A bottle of Agencourt AMPure XP beads (Beckman Coulter, A63881) was warmed to room temperature and gently shaken to resuspend the magnetic beads. 100 μL of beads was added to 200 μL of diluted PCR product (0.5× volumes). Samples were mixed by pipetting and incubated at room temperature for 10 min. Beads were pelleted on a magnet, and the clear solution was transferred to a new tube. Another 30 μL of beads was added to the clear solution (0.65× volume), mixed by pipetting, and incubated at room temperature for 10 min. Keeping the beads on the magnet, samples were washed twice with 200 μL of 70% ethanol (freshly made) without mixing. Ethanol was then completely removed. Beads were left on the magnet for 5 min to allow the remaining ethanol to evaporate. DNA was eluted by adding 20 μL of ddH_2_O, mixing by pipetting, and incubating at room temperature for 5 min. After being separated on a magnet, the solution was transferred to a new tube. DNA was then quantified and sequenced using an Illumina sequencing platform.

### RNA extraction and library preparation

Total RNA was isolated using Quick-RNA MicroPrep Kit (Zymo Research, R1050) according to the manufacturer’s instructions. Then, polyA+ RNA was purified from total RNA by using oligo-dT attached magnetic beads (NEB, E7490), then the purified RNAs were used to prepare RNA-seq library by NEBNext Ultra II Directional RNA Library Prep Kit for Illumina (NEB, E7765) according to the manufacturer’s instructions. Libraries were pooled and sequenced in PE150 mode on the Illumina sequencing platform.

### ChIP-seq library preparation

To depict the CTCF landscape and H3K27ac modification landscape in different developing brain regions, we performed ChIP-seq for the CTCF protein and H3K27ac modifications. Millions of cells were used for each ChIP-seq library construction. Typically, 10–40 million cells were used for a single ChIP experiment. Bulk tissue was washed once with PBS, then centrifuged at 4 °C with speed 800× *g*. To crosslink protein–DNA complexes, 1% formaldehyde was added for 10 min at room temperature. Crosslinking was quenched by adding 125 mM glycine for 5 min at room temperature. Cells were then rinsed three times in ice-cold PBS containing complete protease inhibitor cocktail tablets (Sigma) and collected by scraping. Cells were pelleted and either stored at −80 °C until use or processed immediately. Cell pellets were lysed by buffer 1 (50 mM Tris-HCI, pH 8.0, 10 mM EDTA, pH 8.0, 0.2% SDS, 1 mM PMSF, 20 mM Na-butyrate, and cocktail proteinase inhibitor) for 40 min at 4 °C. Nuclei were then pelleted by centrifugation at 1000× *g*, for 10 min at 4 °C. Sonication was carried out using a BioruptorTM UCD-400 TO set at a HIGH setting of 18 cycles, (30 s on/30 s off), which resulted in genomic DNA fragments with sizes ranging from 200 bp to 2 kb. Insoluble materials were removed by centrifugation at the maximum speed for 10 min at 4 °C. The supernatant was transferred to a new tube. Ten percent of the ChIP sample (10 μL from 100 μL lysate) was saved as input material. The remaining lysate was incubated with antibodies for immunoprecipitation. The antibody incubation was carried out overnight at 4 °C. The beads bound by immune complexes were pelleted and washed three times with each of the following buffers: low-salt buffer (0.1% SDS, 1% Triton X-100, 2 mM EDTA, 20 mM Tris-HCl, pH 8.1, and 250 mM NaCl), high-salt buffer (0.1% SDS, 1% Triton X-100, 2 mM EDTA, 20 mM Tris-HCl, pH 8.1). In each wash, the beads were incubated with wash buffer for 5 min at 4 °C while nutating. The washed beads were then rinsed once with 1× TE buffer (10 mM Tris-HCl, pH 8.0, and 1 mM EDTA). Then, 10 μL Proteinase K was added to each sample, and samples were incubated at 65 °C for 8 h. The immunoprecipitated genomic DNA fragments were then extracted by Beckman XP beads and then back-extracted with water. Libraries were sequenced in PE150 mode on the Illumina sequencing platform. Antibodies: CTCF (A1133, Abclonal) and H3K27ac (ab4729, Abcam).

### Generation and immunostaining of human cortical organoids

Human H9 embryonic stem cells were maintained in Essential 8 Medium (A1517001, Gibco) on 6-well plates coated with Matrigel (354277, Corning). On day 0, we dissociated the target cell colonies into single cells by Accutase (A1110501, Gibco) and suspended them as 100 cells/μL in KSR medium containing DMEM/F-12 (11320082, Gibco), 20% KSR (A3181502, Gibco), 2 mM GlutaMax-I (35050061, Gibco), 0.1 mM NEAA (11140076, Gibco), 0.1 mM beta-mercaptoethanol (21985023, Gibco), 10 μM SB431542(1614, TOCRIS), 0.1 μM LDN-193189 (6032, TOCRIS), and 3 μM endo-IWR1 (3532, TOCRIS). Then we transferred the cells to 96-well V-bottom plates. We replaced half of the medium every day until day 18. On day 18, the medium was replaced with neural induction medium containing DMEM/F12, 1:100 N2 supplement (17502048, Gibco), 2 mM GlutaMax-I, 0.1 mM NEAA, 0.1 μM beta-mercaptoethanol, and organoids were transferred to a 24-well low-cell-adhesion plate. Half of the media was replaced on alternate days.

Organoids were fixed with 4% paraformaldehyde in PBS for 1 h at 4 °C, cryoprotected in 30% sucrose, and embedded in optimal cutting temperature medium. 25-μm cryosections were collected on superfrost slides using a Leica CM3050S cryostat. The slices were blocked with 10% donkey serum in PBS with 0.1% Triton X-100, and then incubated with primary antibodies: PAX6 (901301, BioLegend, 1:200 dilution), TUBB3 (801201, BioLegend, 1:200 dilution). Binding was visualized using an appropriate Alexa Fluor 594 or Alexa Fluor 647 conjugated donkey anti-mouse. Images were collected using an Olympus FV3000 confocal microscope.

### Knockout of SLN gene enhancer

We designed sgRNA to target the putative sequences of *SLN* enhancers using the GPP sgRNA Designer (CRISPRko) (https://portals.broadinstitute.org), then optimized sgRNA design to maximize activity and minimize off-target effects of CRISPR-Cas9. The sgRNA sequences were as follows: SLN sgRNA1 AAG ACC ATT TAA AAC TAC GG, sgRNA2 TAG CTA AGT TAG TCC AGC AA. The knockout sequences were shown in Supplementary Data Source 1. The sgRNAs were cloned into the HP180-CBH-Cas9-CMV-EGFP or HP180-CBH-Cas9-CMV-RFP plasmids. Electroporation was performed using Lonza AMAXA 4D-Nucleofector, and the Human H9 cells expressing the plasmids were cultured for two days. Then the GFP/RFP-double positive cells were detected and isolated into a 96-well plate coated with Matrigel by FACS (one cell each well). Genomic DNA amplification of the target sequence was conducted to confirm the knockout of the enhancer using the following primers: *SLN* forward ACC TGG TAA TAC AGG TGG ACC AT, reverse TGG GAA GGT TCA GAC TCT TGG AAC C.

### Quantitative reverse transcription PCR (qRT-PCR) of target genes

Total RNA samples were isolated from enhancer-knockout cells using the SV Total RNA Isolation System (Z3100, Roche). RNA concentration was measured with a Nanodrop, and RNA integrity was validated by agarose gel electrophoresis. cDNA was prepared using PrimeScriptII 1st Strand cDNA Synthesis Kit (6210, TaKaRa). qRT-PCR was performed on a PCR biosystems QuantStudio 7 Flex instrument (Applied Biosystems) with FS Universal SYBR Green Master (4913914001, Roche). We used the following qRT-PCR primers: *SLN* forward ATG GTC CTG GGA TTG ACT GAG, reverse GTG CCC TCG GAT GGA GAA TG. *GAPDH* (Endogenous Control) forward CCA TGG GTG GAA TCA TAT TGG A, reverse TCA ACG GA TTT GGT CGT ATT GG. The expression level of target genes was normalized to GAPDH and analyzed using ΔΔ^CT^. The experiment was repeated three times independently with similar results.

### Hi–C data processing

The HiCExplorer suite^58–60^ was used for the processing of Hi–C data. Reads were first mapped to the human genome hg19 using bwa mem with parameters ‘-E50 -L0’. Then, all read pairs that were not uniquely mapped (mapping score < 15), dangling end reads, same fragment reads, self-circled reads, self-ligation reads, and other invalid Hi–C reads were discarded. Details about Hi–C data quality were summarized in Supplementary Table S1. After removing duplication, reads were used to generate a raw Hi–C matrix using ‘hicBuildMatrix’. In the raw contact matrix, rows and columns with zero or small total counts were removed because these bins were mostly from repetitive regions. After filtering low-count bins, the matrices were corrected by the ICE method^61^ using ‘hicCorrectMatrix’. The reproducibility for Hi–C data was measured by the GenomeDISCO^62^ reproducibility score. We calculated GenomeDISCO reproducibility score at 200-kb resolution for Hi–C replicates.

### RNA-seq data processing

We used Refseq gene annotation from the UCSC genome browser. Low-quality RNA-seq reads and adapter sequences were removed by Trimmomatic v0.32. Then reads were mapped to the hg19 genome by STAR^63^ with default parameters. Gene expression FPKM was calculated by HOMER software. RNA-seq tracks for visualization were generated by the bamCoverage program in Deeptools2 with parameter ‘–normalizeUsingRPKM’.

### ChIP-seq data processing and peak calling

For ChIP-seq data, the low-quality reads were removed by Trimmomatic v0.32 and then were mapped to the human genome hg19 by bwa mem. Reads with low mapping quality (MAPQ < 10) were filtered, and PCR duplicated reads were removed by Picard tools. The peaks were called by MACS2 with parameters “-g hs -B -p 1e-5 -f BAMPE --SPMR” for narrow peak calling. The tracks of ChIP-seq data (processed with FPKM) were visualized on the WashU Epigenome Browser and IGV Browser. The published fetal brain histone modification and DNase tracks^64^ and ATAC peaks^47,65^^,^ were downloaded from the Roadmap data resource and the GEO database.

### Insulation scores

Insulation score was calculated as described previously^34^ with the public code on Github (*matrix2insulation.pl*; https://github.com/dekkerlab/giorgetti-nature-2016). A sliding 480 kb × 480 kb square along the matrix diagonal was used. The IQR mean signal within the square was then assigned to each 40-kb diagonal bin. This procedure was then repeated for all 480-kb diagonal bins. The insulation score was normalized relative to all of the insulation scores across each chromosome by calculating the log2 ratio of each bin’s insulation score vs the mean of all insulation scores. Valleys/minima along the normalized insulation score vector represent the loci of reduced Hi–C interactions that occur across the bin. These valleys/minima are interpreted as areas of high local insulation.

### A/B compartments, TADs, and chromatin Loops

We used HOMER^66^ software with parameters ‘–res 100,000, -superRes 400,000’ to obtain the PC1, PC2, and PC3 values. Because sometimes PC2 or PC3 value reflects A/B compartments, we manually checked of PC1, PC2, and PC3 tracks with gene density and the plaid pattern in the correlation heatmaps along each chromosome and got the final ‘PC1’ list. The direction of the Eigen (PC) values is arbitrary, and therefore positive values were set to ‘A’ and negative values were set to ‘B’ based on their association with gene density. The Spearman’s correlation between the A/B compartment PC1 value and DNase chromatin accessibility signal/histone modification signal enrichment was calculated at 100-kb bin resolution. We identified A/B compartment dynamics regions as statistically significant variability in PC1 values across developmental stages using ANOVA with adjusted *P* value < 0.05.

TADs and TAD boundaries were identified based on the public TAD separation score method^58–60^ with the ‘hicFindTADs’ command in the HiCExplorer suite. The TAD-separation scores in human embryos were calculated at 40-kb resolution using parameters “--minDepth 300,000 –maxDepth 3,000,000 --step 300,000 --minBoundaryDistance 400,000 –thresholdComparisons 0.01 –correctForMultipleTesting fdr –delta 0.01”. The regions with *q* < 0.01 were reported as boundaries. TADs were identified as regions between two boundaries. Differential Domains were identified by the ‘Diffdomain’ software.

We applied ‘hicDetectLoops’^58–60^ to detect enriched/significant interaction peaks within chromosomes at 10-kb resolution. Briefly, for each genomic distance, a negative binomial distribution is computed, and only interaction bin pairs with *P* value < 0.05 are accepted as candidate loops. Next, each candidate is compared to its neighborhood. This neighborhood is defined by the window size in the *x* and *y* dimensions around the candidate. Per neighborhood, only one candidate is considered, therefore, only the candidate with the highest peak values is accepted. We performed a parameter sweep for the chromatin loops and merged all loops into the final loop annotation list.

For a given gene such as *SLN* or a brain disease-associated SNP, we also applied the method introduced in Won et al.^7^ to identify significant Hi–C interactions. Briefly, to avoid significant Hi–C interactions affecting the distribution fitting, as well as parameter estimation, the lowest 95 percentiles of Hi–C contacts were used, and zero contact values were also removed. Using these background Hi–C interaction profiles, the distribution of Hi–C interactions at each distance for each chromosome was determined using the fitdistrplus package. The Weibull distribution was used to calculate the statistical significance for a given Hi–C interaction at the matched chromosome and distance. Hi–C contacts with *P* value < 0.0001 were selected as significant interactions. The interaction profile for a given locus was visualized by the Gviz package.

### Feature-free chromatin structure difference detection for different brain regions

To find out the dynamics of chromomatin 3D structures in different human brain regions, we employed a feature-free method CHESS^40^, to detect differences. CHESS can robustly identify and classify specific similarities or differences and features in chromatin contact data like Hi–C data. CHESS applies the structural similarity index (SSIM), which was widely used in image analysis to chromatin contact matrices, assigning a structural similarity score and an associated *P* value to pairs of genomic regions. Briefly, to calculate the similarity between a reference (*R*) and query (*Q*) matrix, their entries were first divided by the expected contact intensity at the respective distance to remove the distance dependency of pairwise contact probabilities characteristic of Hi–C matrices. CHESS then scaled the matrices to equal size and calculated the SSIM between *R* and *Q*. The SSIM score (*S*) was a single value, combining brightness, contrast, and structure differences between two matrices. Brightness was calculated as the mean of the signal intensity. Contrast was calculated as the variance in signal. The structure term was calculated as the correlation between the signal values of two matrices. S was then defined as a weighted product of these three components, which were scaled such that S ranged between −1 (inversion) and 1 (identity), where *S* = 0 indicates no similarity. *S* can be used directly to quantify changes in chromatin contacts within windows of a given size across the genome: for identical matrices, *S* = 1, and the lower the score, the larger the change. CHESS version 0.3.7 was run using window sizes of 2 Mb, with a 500-kb step size, for data binned at 25-kb resolution, and 4 Mb windows with a 1 Mb step size for data binned at 50-kb resolution.

### The dynamic gene expression clusters during brain development

To find out how the time-course gene expression dynamics occur during the multiple human brain region development, we filter out the unexpressed genes (FPKM < 0.5) in all stages and use the ‘TCseq’ package to capture the temporal patterns of the transcriptome by soft clustering. The genes with membership > 0.5 for each cluster were used for GO enrichment analysis by the ‘clusterProfiler’ package.

### SEs

All H3K27ac ChIP-seq data sets were aligned using BWA (version 0.7.15) mem to build version hg19 of the human genome. We randomly downsampled 35 million reads from treated and control groups in each sample, and then used them to call peaks by MACS2. We used ROSE^67^ software to identify SEs. In order to accurately capture dense clusters of enhancers, we allowed regions within 12.5 kb of one another to be stitched together and excluded regions contained within +/−2500 kb from TSS in order to account for promoter biases. Only if the number of constituents stitched was more than 2, the SEs were considered to be valid and were used for further processing.

### GWAS SNP analysis and identification of target genes

GWAS SNPs for a total of 8 neuropsychiatric disorders or mental traits (ASD, SCZ, ADHD, MDD, AD, PD, ALS, and IQ) were mined from the GWAS Catalog (September 2019) using a *P* value threshold of 10^−6^ (details in Supplementary Table S5). The GWAS SNPs were expanded to sets of linked SNPs using HaploReg 4.1 at an LD threshold of 0.8 according to the reported study population(s) for each SNP. All SNPs were fitted to hg19 and filtered for duplicates by position. If genes have significant interactions/loops with the GWAS SNPs in a certain human brain region, we identified the genes as GWAS SNP-linked genes.

### Statistics and reproducibility

R and Prism were used for statistical analysis. Wilcoxon rank sum test was used for statistical significance (Fig. 3c and Supplementary Fig. S2c) with the Wilcox test function in R. Two-sided unpaired *t*-test was used for statistical significance (Fig. 4f, h and Supplementary Fig. S10g). The GO functional enrichment was calculated by the ‘clusterProfiler’ package and the DAVID. For violin plots, the white dots represent the median values, boxes and whiskers represent the 25th/50th/75th percentiles and 1.5× the interquartile range, respectively. *P < 0.05, **P < 0.01, ***P < 0.001, ****P < 0.0001, n.s., no significance. All of the statistical details can be found in the figure legends and methods.

## Supplementary information

Supplementary Information

Supplementary Data Source 1

Supplymentary Table S1

Supplymentary Table S2

Supplymentary Table S3

Supplymentary Table S4

## Data Availability

The accession number for the raw sequencing data reported in this paper is GSA for human: HRA002848. Alternatively, the data can also be visualized on the WashU Epigenome Browser (http://epigenomegateway.wustl.edu/browser/?sessionFile=https://usa-3dg.oss-us-west-1.aliyuncs.com/washuSession/eg-session-YDR8Em48Q-47483d90-88a9-11ec-bf97-87e935016735.json) (session ID: 47483d90-88a9-11ec-bf97-87e935016735). Tracks include CTCF ChIP-seq and H3K27ac ChIP-seq for different developing brain regions. Raw image files used in the figures that support the findings of this study are available from the corresponding authors upon reasonable request.

## Code availability

All analyses in our study were performed with open-source packages as described in the “MATERIALS AND METHODS” section. All relevant codes are available from the corresponding authors upon reasonable request.
