# Supplementary material for: Spatiotemporal 3D chromatin organization across multiple brain regions during human fetal development
Source: Cell Discov. 2025 May 16;11:50. doi: 10.1038/s41421-025-00798-w (PMC12081887; doi:10.1038/s41421-025-00798-w)
Supplement: Supplementary file 1 — Supplementary Information [file 41421_2025_798_MOESM1_ESM.pdf]

Spatiotemporal 3D chromatin organization of multiple brain regions during the fetal development in human

Supplementary materials including:

Supplementary figures and legends

Materials and methods

Caption for Supplementary Table S1-4

Caption for Supplementary Data Source 1

Other supplementary material for this manuscript includes:
Supplementary Table S1-4 (Excel format)

Supplementary figures and legends

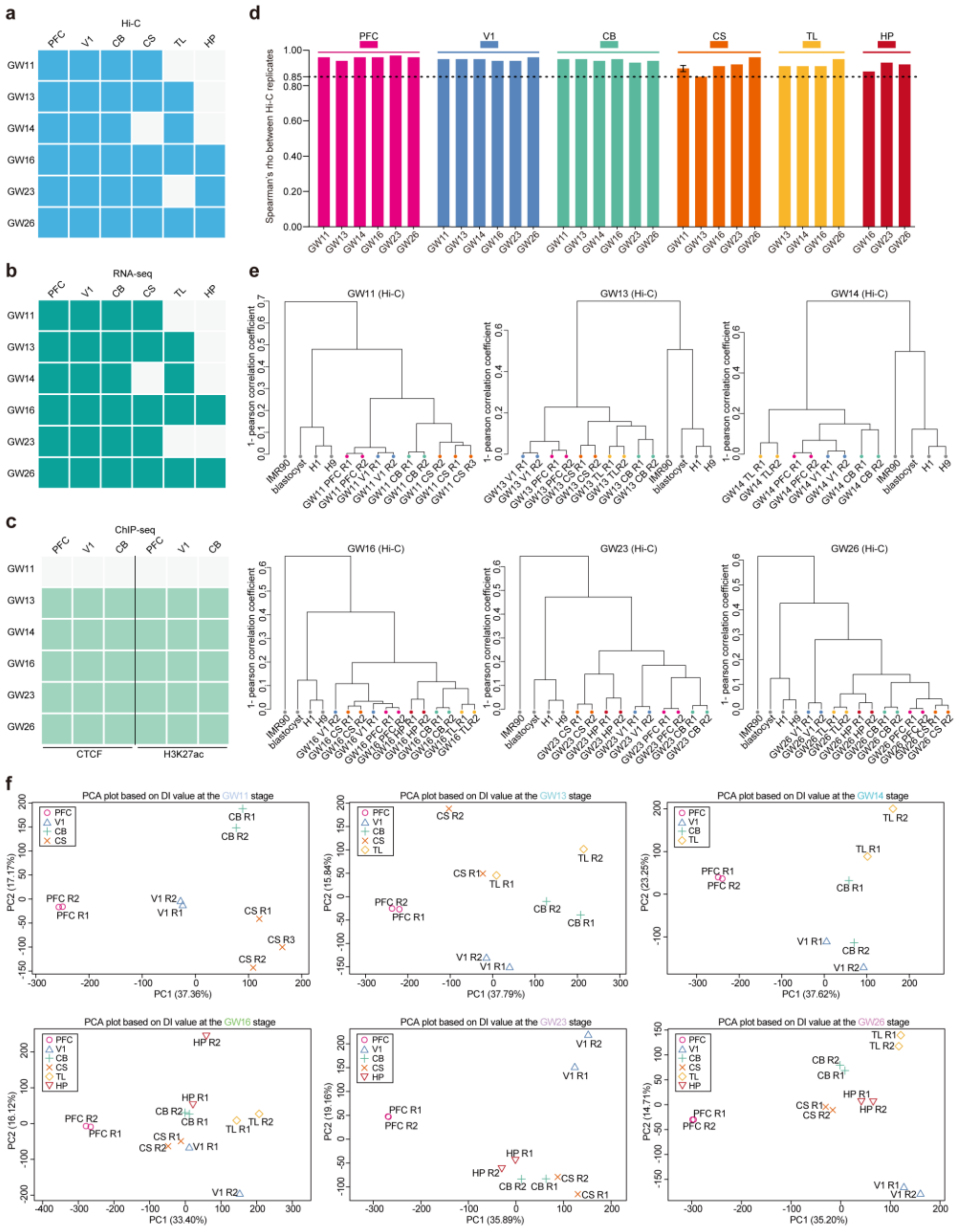

Supplementary Fig. S1: 3D chromatin landscapes in multiple developing human brain regions.

(a-c) The sample summary for 3D epigenome experiments in this study. (**a**) Hi-C experiments, (**b**) RNA-seq experiments and (**c**) ChIP-seq experiments. Grey boxes mean no data sequenced in this study.

(d) Barpots for the Hi-C replicate reproducibility in developing human PFC, V1, CB, CS, TL and HP regions at different stages.

(e) The hierarchical clustering of developing brain regions at different developmental stages along with published IMR90 cells, H1 ESCs, H9 ESCs and human blastocysts based on A/B compartment PC1 values.

(f) The PCA plot of developing brain regions at different developmental stages based on Directional Index (DI) values.

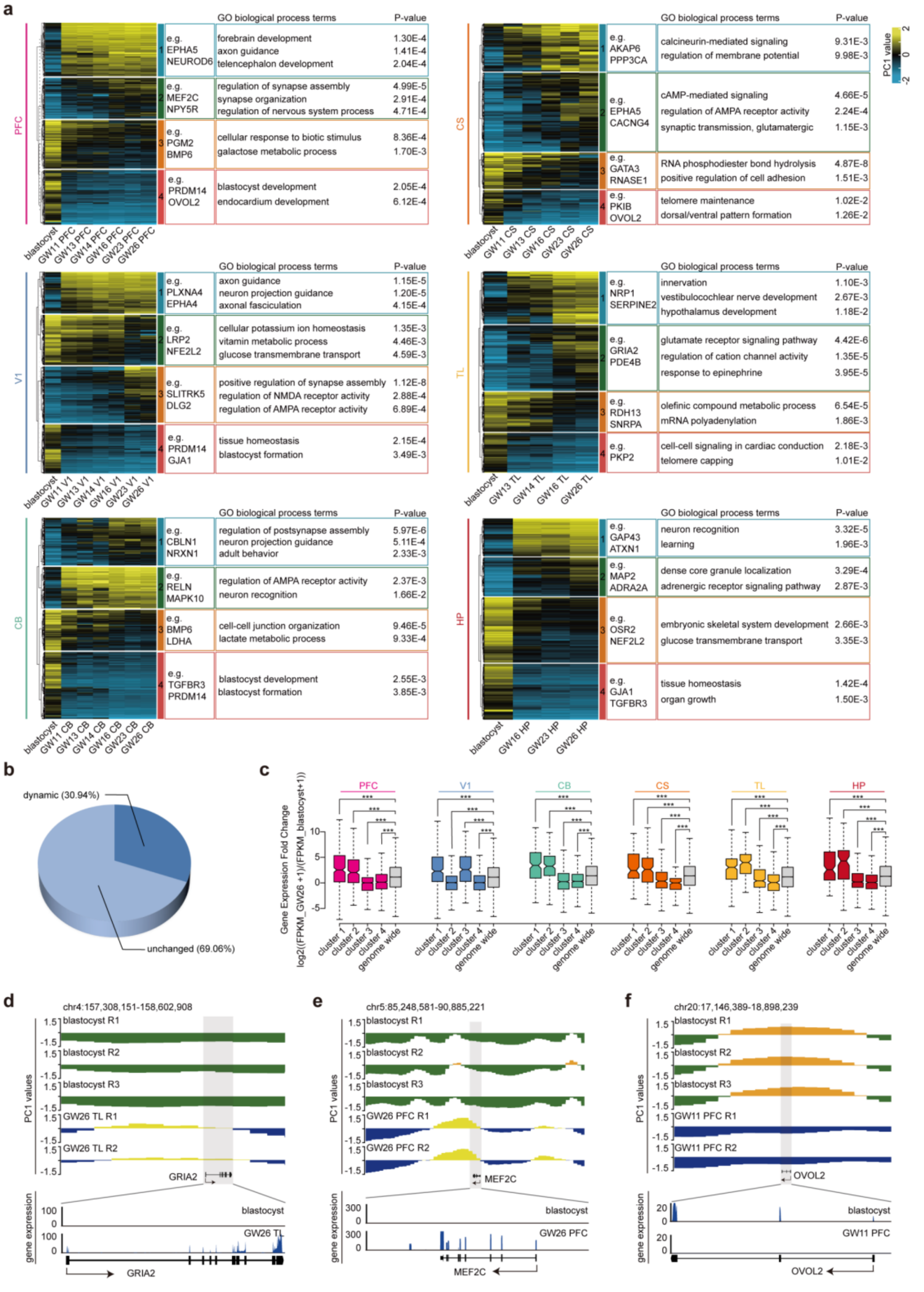

Supplementary Fig. S2: Dynamic A/B compartments during the human brain development.

(a) A/B compartment PC1 value heatmap for A/B compartment switched regions and GO enrichment results for genes with A/B compartment switches in human blastocysts and multiple human brain regions.

(b) Pie chart for the proportion of dynamic and unchanged A/B compartment switched regions.

(c) Boxplots of gene expression fold change for genes with A/B compartment switches in multiple developing brain regions.

(d-f) Snapshots for the A/B compartment switches and gene expressions of *GRIA2* gene (**d**), *MEF2C* gene (**e**) and *OVOL2* gene (**f**).

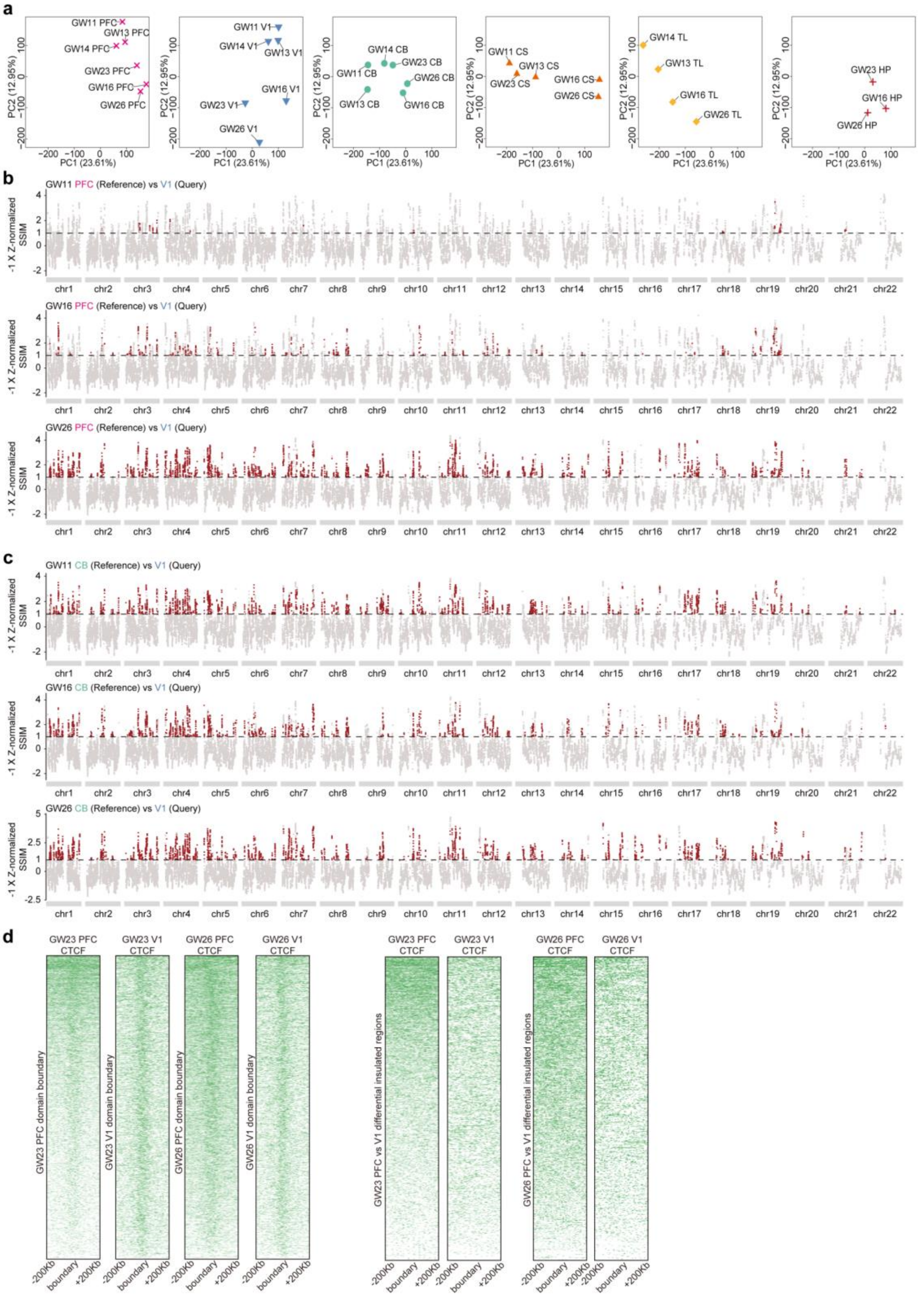

Supplementary Fig. S3: The dynamic 3D chromatin organization across multiple human brain regions.

(a) 2D PCA plots for PFC, V1, CB, CS, TL and HP samples based on the insulation score according to the PC1 and PC2 values. Proportions of variance

explained by the PC1 and PC2 values were also shown.

(b) The distribution of dissimilar genomic regions between the PFC region and V1 region across the whole genome at the GW11 (top), GW16 (middle) and GW26 (bottom) stages. Dissimilar genomic regions were marked in red.

(c) The distribution of dissimilar genomic regions between the CB region and V1 region across the whole genome at the GW11 (top), GW16 (middle) and GW26 (bottom) stages. Dissimilar genomic regions were marked in red.

(d) Heatmaps for CTCF binding signals along domain boundaries and differential insulated regions in the GW23PFC/GW23V1 and GW26PFC/GW26V1.

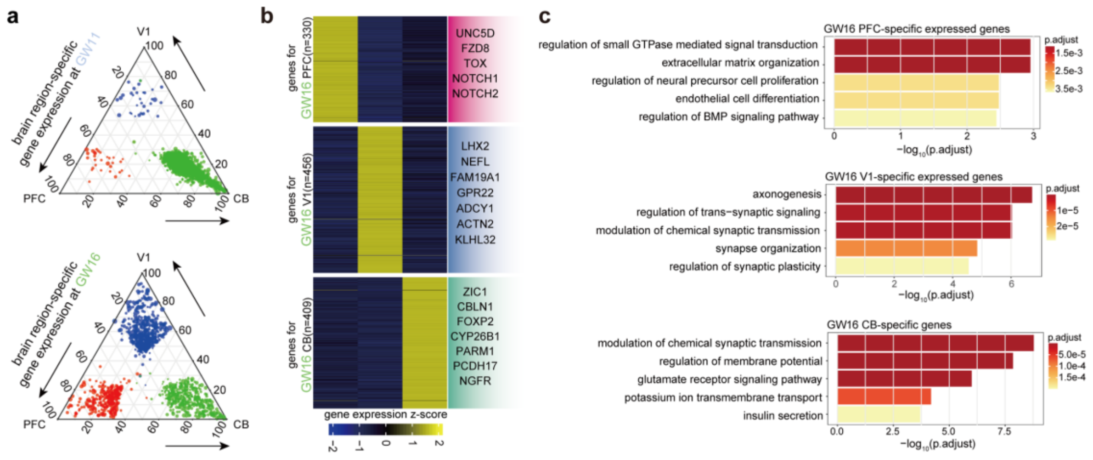

Supplementary Fig. S4: The dynamic gene expressions for PFC, V1 and CB regions.

(a) Ternary plots of brain region-preferentially expressed genes at the GW11 stage (top) and at the GW16 stage (bottom).

(b) Heatmap of gene expression for brain region-preferentially expressed genes in the GW16 PFC, V1 and CB regions.

(c) Barplots of GO biological process enrichment for brain region-preferentially expressed genes in the GW16 PFC, V1 and CB regions.

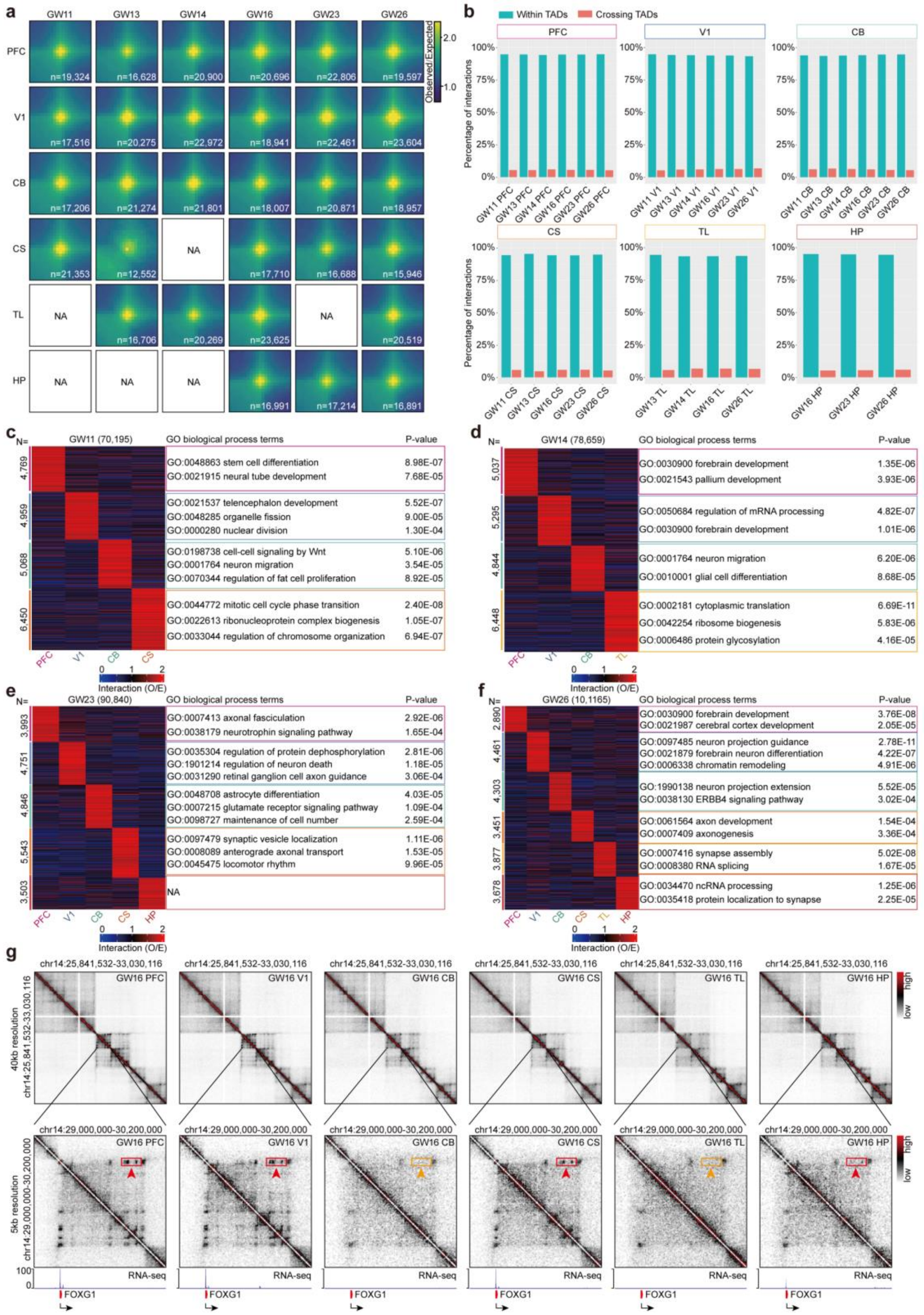

Supplementary Fig. S5: Brain region specific chromatin loops in developing human brains.

(a) The chromatin loops for multiple developing human brain regions. Loop numbers (n) were also shown. 'NA' represents no data in this study.

(b) The percentage for loops within TADs and for loops crossing TADs in human PFC, V1, CB, CS, TL and HP regions.

(c-f) O/E value heatmaps for brain region specific chromatin loops and their associated gene function enrichments at the GW11 stage (**c**), the GW14 stage (**d**), the GW23 stage (**e**) and the GW26 stage (**f**). GW23 HP-specific loops without gene expression data in the GW23 HP sample (**e**).

(g) The 40kb-resolution interaction heatmaps at the GW16 stage and zoom-in 5kb-resolution heatmaps around the *FOXG1* overlaid with *FOXG1* gene expression.

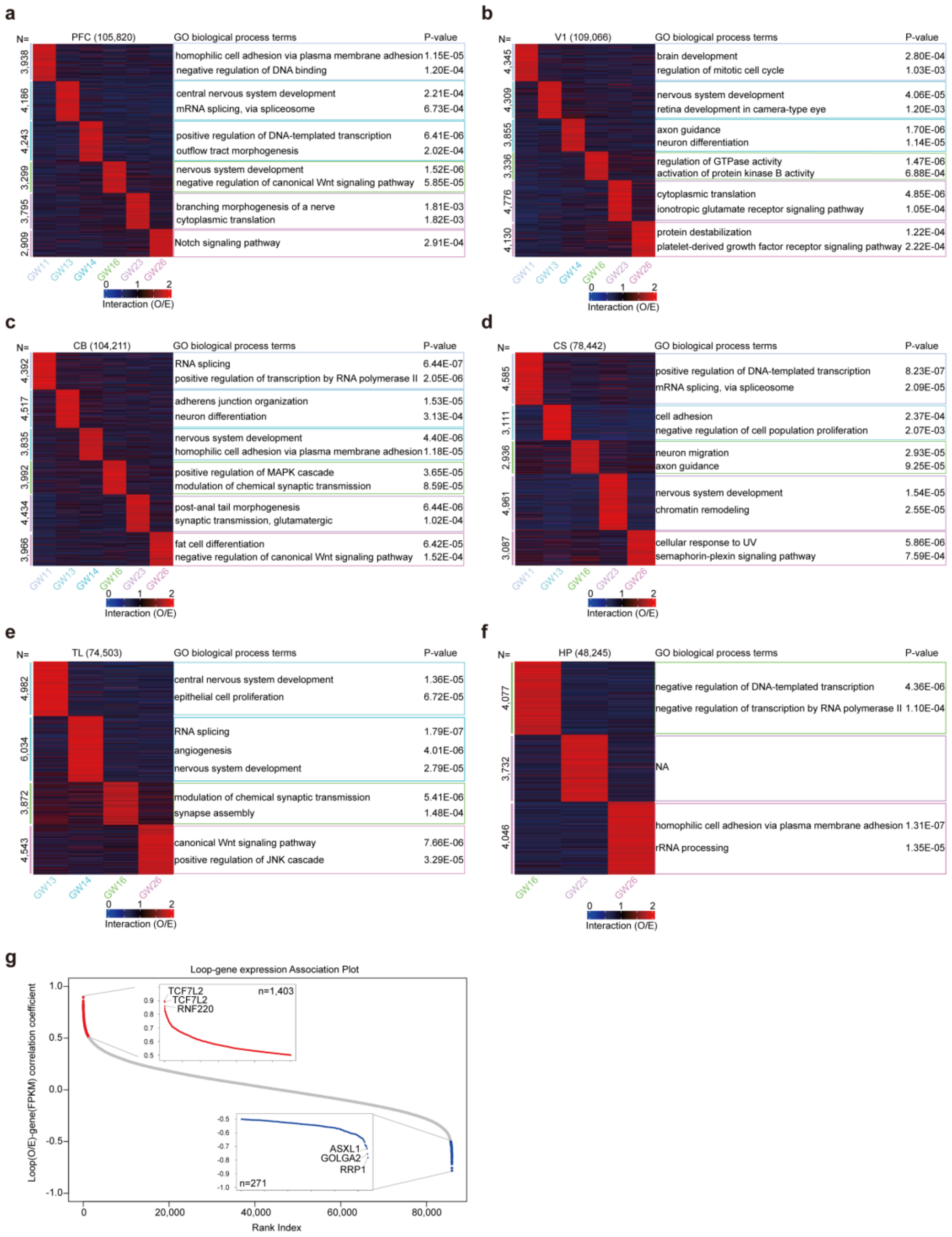

Supplementary Fig. S6: Developmental stage specific chromatin loops in developing human brains.

(a-f) O/E value heatmaps for developmental stage specific chromatin loops and their associated gene function enrichments in the PFC region (**a**), the the V1

region (**b**), the CB region (**c**), the CS region (**d**), the TL region (**e**) and the HP region (**f**). GW23 HP-specific loops without gene expression data in the GW23 HP sample (**f**).

(g) Sorted scatter plot for loop-gene pairs. Red dots represents significantly positively correlated loop-gene pairs (correlation>0.5) and blue dots represents significantly negatively correlated loop-gene pairs (correlation<-0.5).

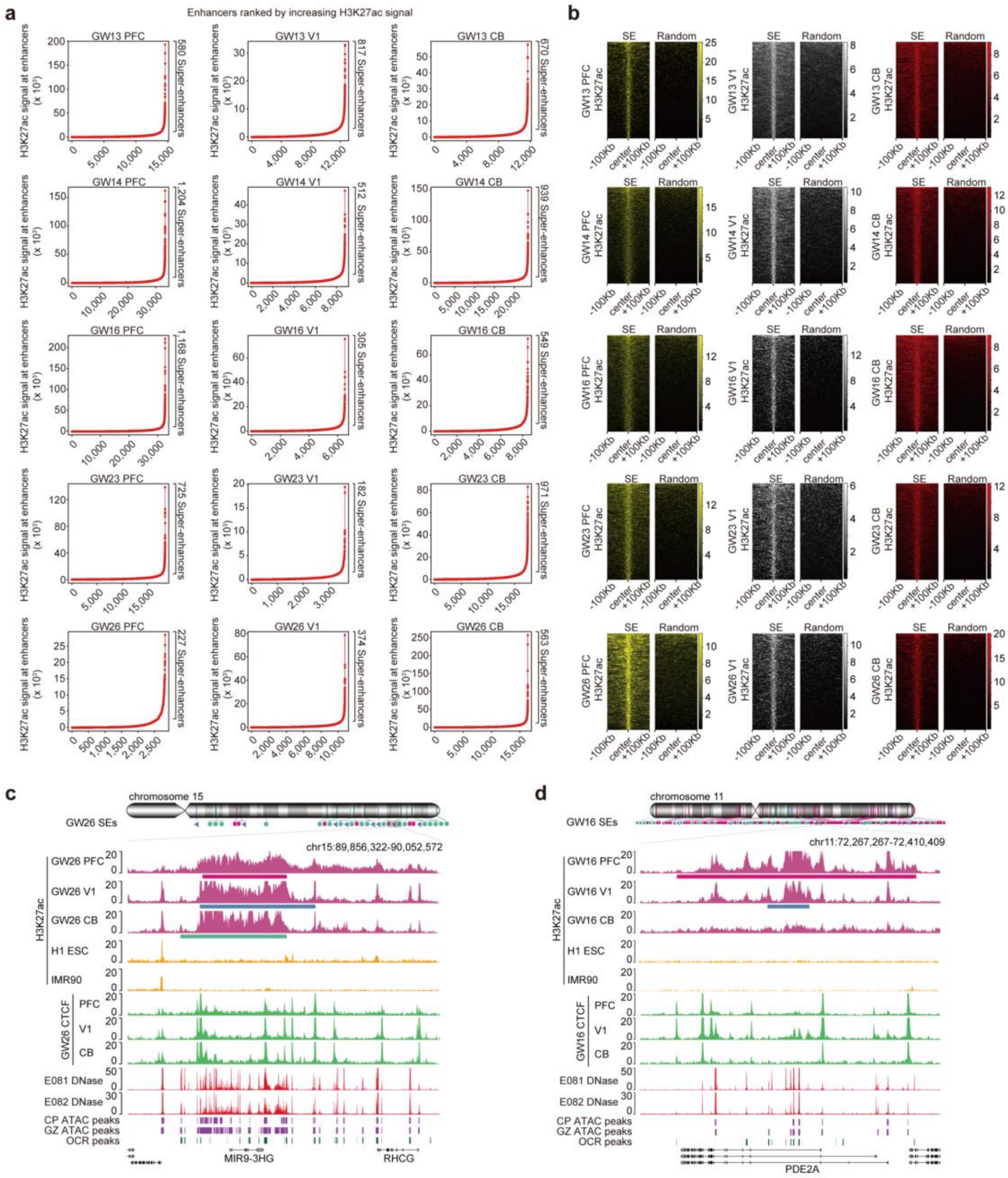

Supplementary Fig. S7: Super enhancers (SEs) in multiple developing brain regions.

(a) The accumulative H3K27ac signal for super enhancers identified in different human brain regions.

(b) H3K27ac signal enrichment heatmaps around super enhancers and random controls in different human brain regions.

(c) An super enhancer in human PFC, V1 and CB regions around *MIR9-3HG* gene but not for H1 ESC and IMR90 cells, overlaid with H3K27ac ChIP-seq tracks, CTCF ChIP-seq tracks and published chromatin accessibility tracks (Roadmap E081/E082 DNase-seq tracks, CP/GZ ATAC peaks (GSE95023) and brain Open Chromatin Region peaks (OCR peaks, GSE149268)).

(d) Differential super enhancers in the PFC/V1 region but not in the CB region around the *PDE2A* gene overlaid with H3K27ac ChIP-seq tracks, CTCF ChIP-seq tracks and published chromatin accessibility tracks (Roadmap E081/E082 DNase-seq track, CP/GZ ATAC peaks and brain OCR peaks).

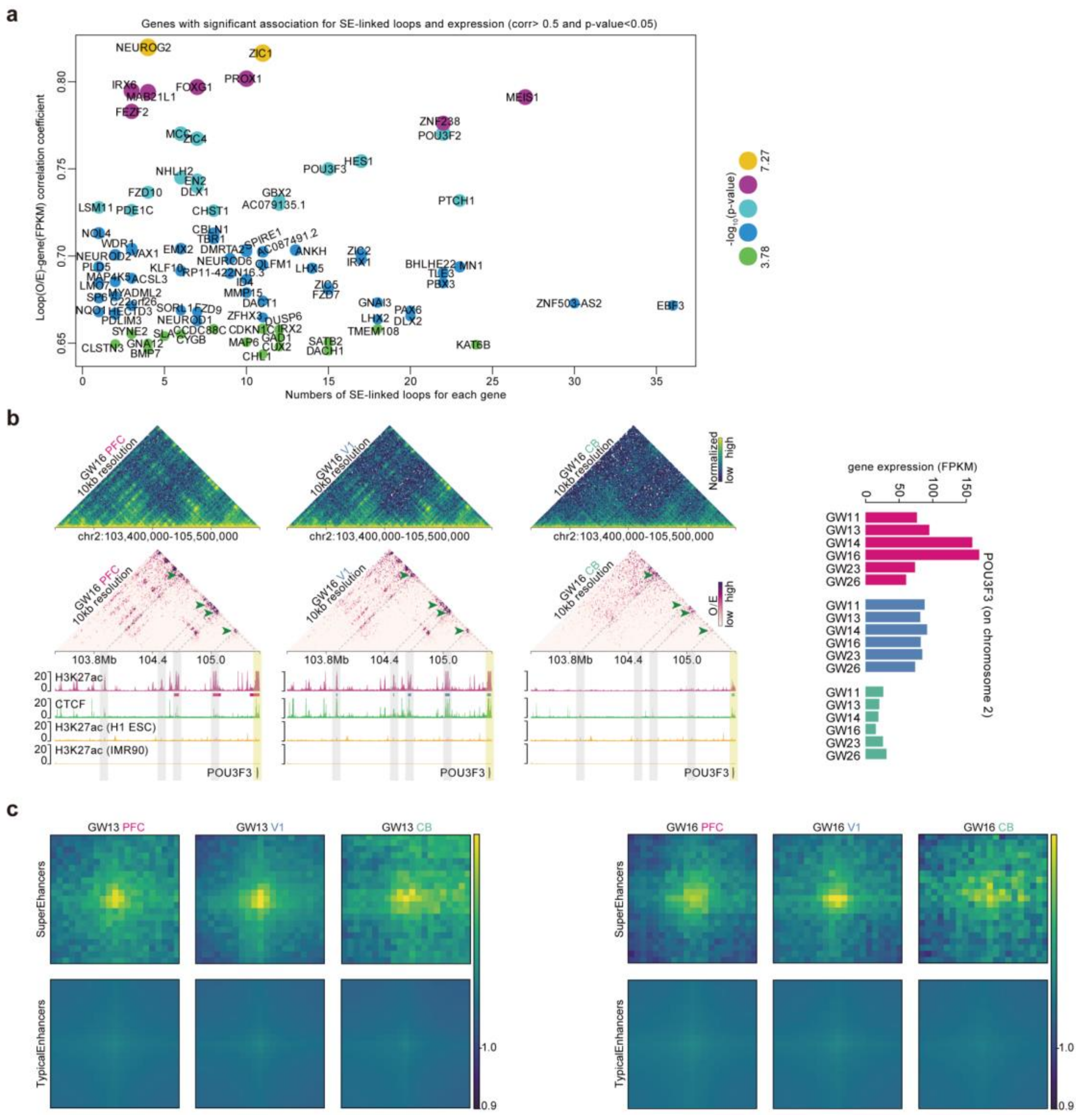

Supplementary Fig. S8: SE-associated loops in the human developing brain.

(a) The scatter plot for 89 genes with significant association for SE-linked loops and expression (corr> 0.5 and p-value<0.05). The color of each dot represents the correlation significance $-\log_{10}$(p-value). The Loop(O/E)-gene(FPKM) correlation coefficients were calculated based on the pearson correlation test from the loop O/E value vector and the expression vector of their promoter-linked genes.

(b) 10kb-resolution O/E interaction heatmaps around POU3F3 gene overlaid with brain H3K27ac ChIP-seq tracks, CTCF ChIP-seq and H1/IMR90 H3K27ac ChIP-seq tracks. Green arrows indicated dynamic loops mediated by H3K27ac-marked SEs in the PFC/V1 region (left panel). The *POU3F3* gene expression barplot of PFC, V1 and CB samples from the GW11 to GW26 stage (right panel).

(c) The enrichment plot for interactions between identified SEs and interactions between typical enhancers in the PFC, the V1 and the CB samples.

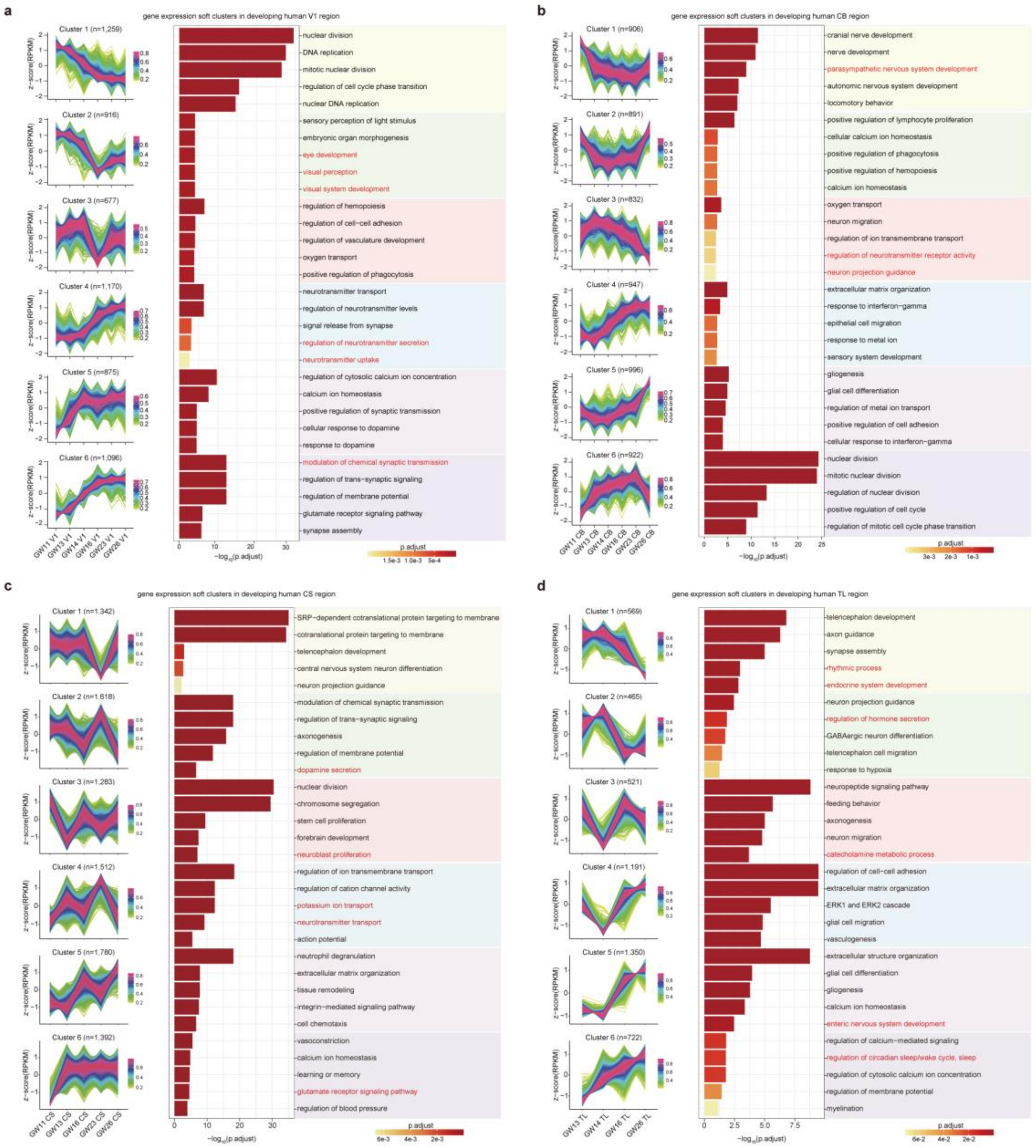

Supplementary Fig. S9: The dynamic gene expression clusters during the development of multiple brain regions.

(a-d) The dynamic soft clustering of genes during the development of multiple brain regions and their GO biological process enrichment results. (**a**) for the V1 region, (**b**) for the CB region, (**c**) for the CS region and (**d**) for the TL region

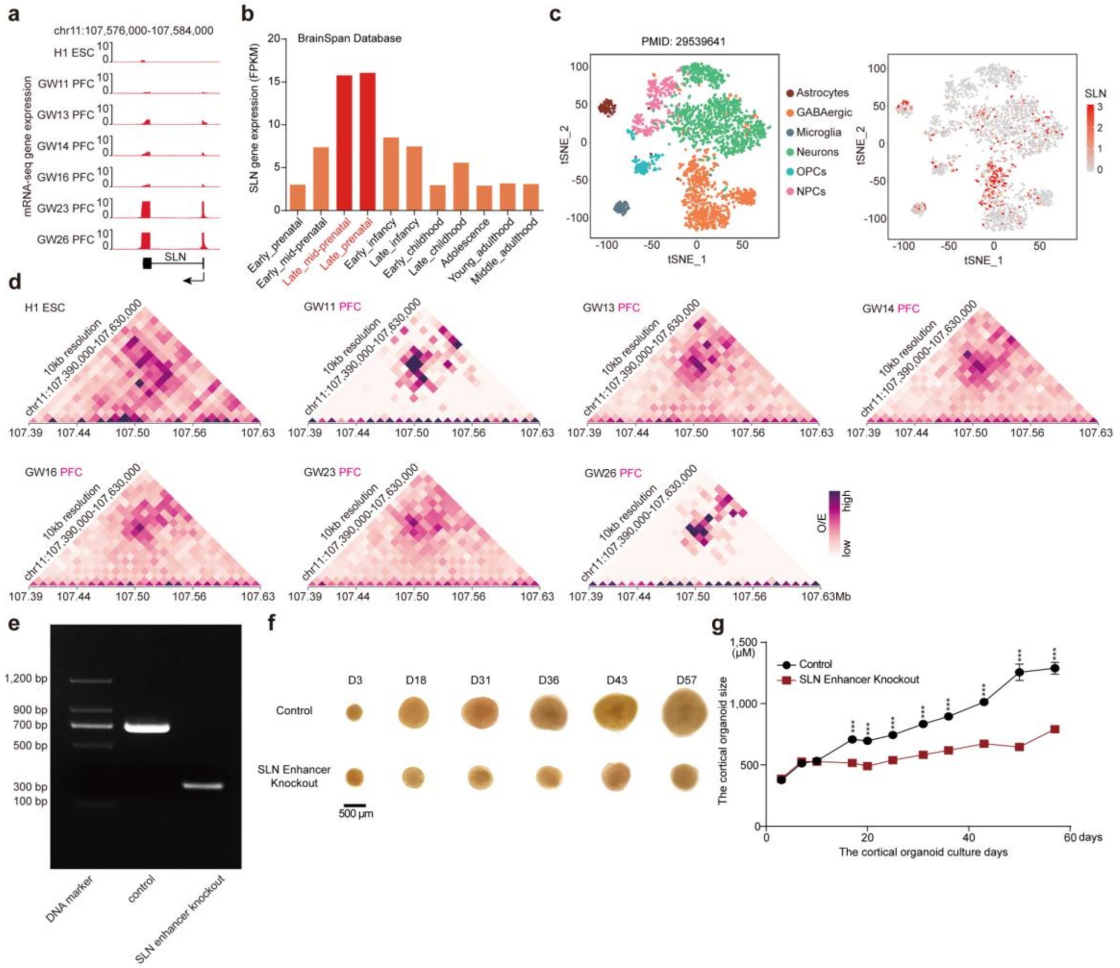

Supplementary Fig. S10: The knockout of SLN's enhancer could reduce the neuron maturation.

(a) The dynamic *SLN* gene expression tracks from H1 ESCs to GW26 PFC.

(b) The *SLN* gene expression dynamics for 11 different developmental stages from the public BrainSpan database.

(c) The *SLN* gene expression in different cortical cell types. Single-cell RNA data was from GSE104276 (PMID: 29539641).

(d) O/E interaction heatmaps for the SLN gene to enhancer in the H1 and GW11 PFC to GW26 PFC.

(e) The DNA electrophoretic gel picture for the SLN enhancer knockout and the control along with DNA marker.

(f) The human cortical organoid development for control and SLN enhancer knockout samples. Scale bar, 500 μm. D3, i.e. Day 3.

(g) The organoid size for control and SLN enhancer knockout samples during cortical organoid development. Error bars represent mean±s.e.m.. *** p.value < 0.001 (two-sided t.test).

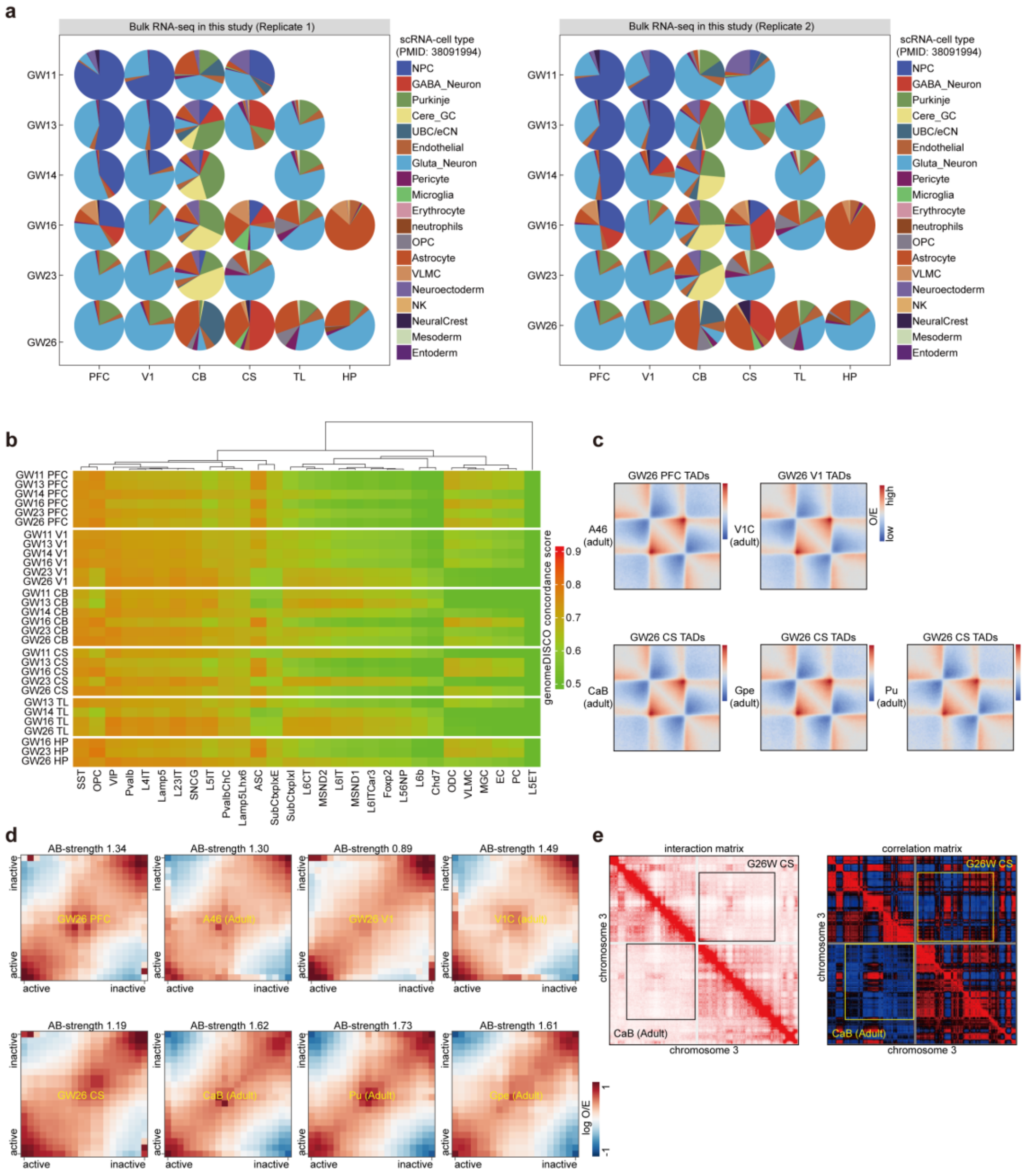

Supplementary Fig. S11: The investigation of virtual cell types in developing human brains.

(a) The pie charts for the deconvolution results of our bulk RNA-seq with single-cell transcriptome in the developing human brain (PMID: 38091994).

(**b**) The Pearson correlation heatmap for our Hi-C datasets from each GW stage to the published single-cell adult human brain Hi-C data (PMID: 37824674).

(c) TAD enrichment heatmap for adult brain regions onto the developing brain regions. A46 is for Middle frontal gyrus from cerebral cortex. V1C is for Primary Visual Cortex from cerebral cortex from cerebral cortex. CaB, Pu and Gpe are belong to corpus striatum from Basal nuclei (Body of the Caudate, CaB; Putamen, Pu; Globus pallidus, Gpe).

(d) The A/B compartment saddle plots and their AB-compartment strength (AA * BB / AB^2) for adult brain regions and corresponding GW26 brain regions

(e) An example showing the interactions across A-B compartments on chromosome 3. The left panel is for normalized interaction matrix and the right panel is for correlation matrix.

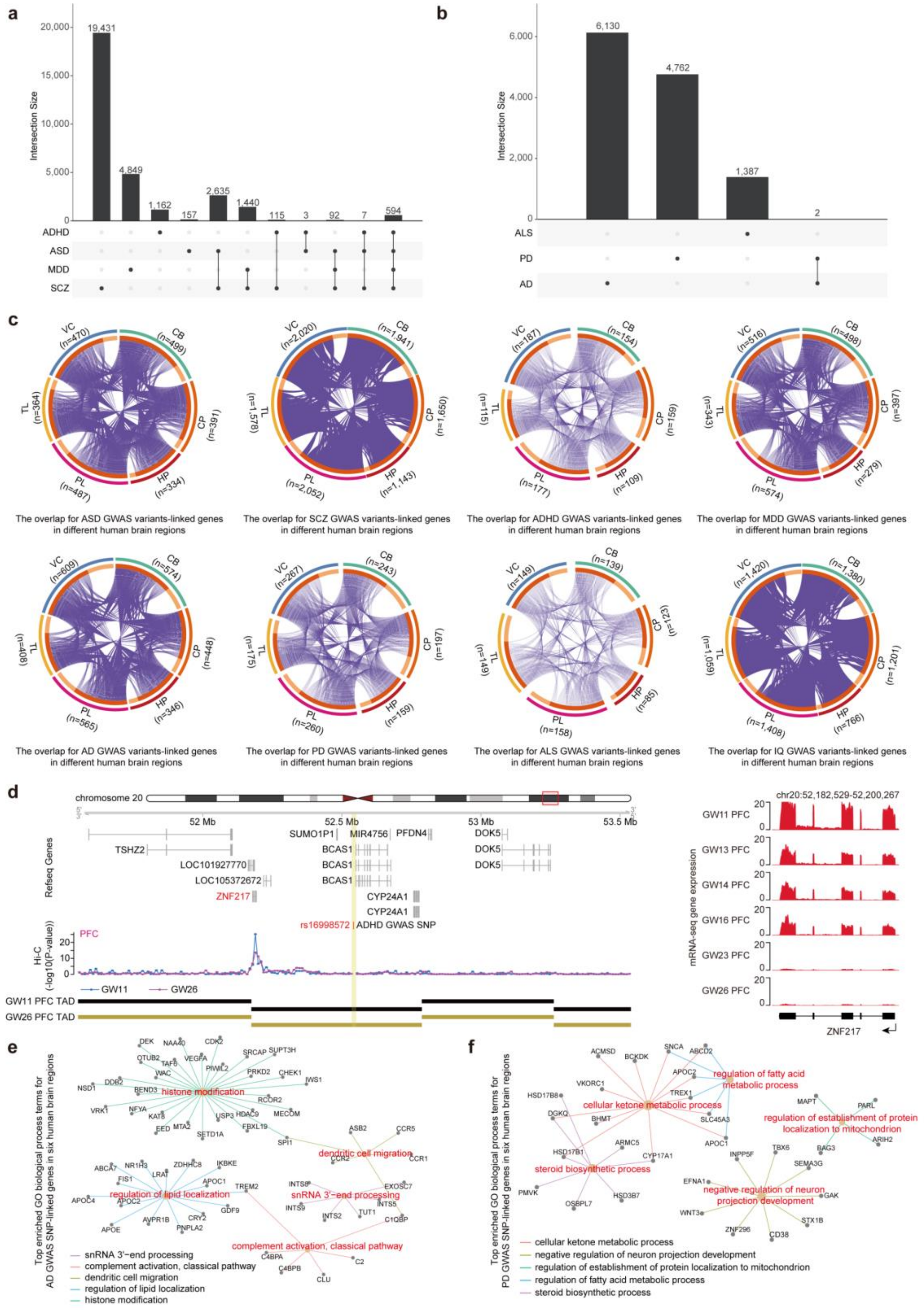

Supplementary Fig. S12: The SNP-linked genes for neuropsychiatric GWAS SNPs.

(a) The upset plot for GWAS SNPs of developmental nervous and mental diseases.

(b) The upset plot for GWAS SNPs of neurodegenerative diseases.

(c) Circular overlap plots for GWAS SNP-linked genes in different brain regions for ASD, SCZ, ADHD, MDD, AD, PD, ALS and IQ, respectively.

(d) The interaction tracks of an ADHD GWAS SNP rs16998572 in the PFC region. The ADHD GWAS SNP rs16998572 and its linked gene *ZNF217* were marked in red.

(e) The top enriched GO term-gene networks of AD GWAS SNP-linked genes identified from the total six brain regions.

(f) The top enriched GO term-gene networks of PD GWAS SNP-linked genes identified from the total six brain regions.

Materials and Methods

No statistical methods were used to predetermine sample size. The experiments were not randomized and investigators were not blinded to allocation during experiments and outcome assessment.

Human ethics statement

The regulatory framework about the use of human fetal brain samples for this research was based on the policies of the Human Biomedical Research Ethics Guidelines (set by National Health Commission of the People's Republic of China on Dec. 1st ,2016), the 2016 Guidelines for Stem Cell Research and Clinical Translation （issued by the International Society for Stem Cell Research, ISSCR） and the Human Embryonic Stem Cell Research Ethics Guidelines (set by China National Center for Biotechnology Development on Dec.24, 2003). All the protocols are in compliance with the 'Interim Measures for the Administration of Human Genetic Resources' administered by the Ministry of Science and Technology of China.

The human tissue collection and research protocols were approved by the Reproductive Study Ethics Committee of Beijing Anzhen Hospital (2014012x) and the Institutional Review Board of Institute of Biophysics (H-W-20131104). All fetal brain samples were collected under standard clinical protocols after the donor patients signed an informed consent document. No statistical methods were used to predetermine sample size.

Data availability

The accession number for the raw sequencing data reported in this paper is GSA for human: HRA002848. Alternatively, the data can also be visualized on the WashU Epigenome Browser (http://epigenomegateway.wustl.edu/browser/?sessionFile=https://usa-3dg.oss-us-west-1.aliyuncs.com/washuSession/eg-session-YDR8Em48Q-

47483d90-88a9-11ec-bf97-87e935016735.json) (session ID: 47483d90-88a9-11ec-bf97-87e935016735). Tracks include CTCF ChIP-seq and H3K27ac ChIP-seq for different developing brain regions. Raw image files used in the figures that support the findings of this study are available from the corresponding authors upon reasonable request.

Fetal brain sample collection and dissection

De-identified fetal brain tissues with no karyotype abnormalities or genetic conditions reported were collected in fresh ice-cold artificial cerebrospinal fluid containing 125.0 mM NaCl, 26.0 mM $NaHCO_3$, 2.5 mM KCl, 2.0 mM $CaCl_2$, 1.0 mM $MgCl_2$, 1.25 mM NaH_2PO_4 at a pH of 7.4 when oxygenated (95% O_2 and 5% CO_2).

For different fetal brain regions, Microdissections were performed carefully to sample target regions, including cerebral prefrontal cortex (PFC), primary visual cortex (V1), cerebellum (CB), subcortical corpus striatum (CS), thalamus (TL) and hippocampus (HP). The collected fetal brain tissue was dissected and placed in hibernate E medium (Invitrogen, A1247601), and then different brain region samples were stored in liquid nitrogen for subsequent experiments.

In situ Hi-C library generation for human fetal brain samples

In situ Hi-C libraries for samples from different human fetal brain regions were performed according to previous protocols[58]. Concretely, brain sample was fixed with 1 ml of freshly made 1% formaldehyde solution and incubated at RT for 10 min. To quench the reaction, 2.5 M glycine solution was added to a final concentration of 0.2 M. Samples were incubated at room temperature for 5 min and then centrifuge for 5 min at 3000 Xg at 4 °C. Discard supernatant. The pellet was washed with ice-cold 1x PBS and spin for 5 min at 3,000 Xg at 4°C. Discard supernatant and flash-freeze cell pellets in liquid nitrogen. Either proceed to the rest of the protocol or store cell pellets at -80 °C. The sample pellet was resuspend with 1 ml of ice-cold Hi-C lysis buffer (10 mM Tris-HCl pH

8.0, 10 mM NaCl, 0.2% Igepal CA630) with a protease inhibitor cocktail. The cells incubate on ice for 30 min. Samples were then centrifuged at 3,000 Xg for 5 min and the supernatants carefully discarded. Pelleted nuclei were washed once with 1ml of ice-cold Hi-C lysis buffer. Discard the supernatant and resuspend the pellet with 50 μL of 0.5% sodium dodecyl sulfate (SDS). And incubated at 62 °C for 10 min. After incubating, 145 μL of water and 25 μL of 10% Triton X-100 were added to quench the SDS. Tubes were gently tapped to mix well, avoiding excessive foaming and then incubated at 37 °C for 15 min. 25 μL of 10X NEBuffer 2 and 100 U of MboI restriction enzyme (NEB, R0147) were added and chromatin was digested at 37 °C for overnight with rotation. Samples were incubated at 62 °C for 20 min to inactivate MboI and then cooled to RT. To fill in the restriction fragment overhangs and mark the DNA ends with biotin, 50 μL of fill-in master mix (37.5 μL of 0.4 mM biotin-14-dATP, 4.5 μL of 10 mM dCTP/ dGTP/ dTTP mix, 8 μL of 5 U/μL DNA polymerase I, large) was added. Samples were mixed by pipetting and incubated at 37 °C for 1.5h. Ligation master mix (663 μL of water, 120 μL of 10X NEB T4 DNA ligase buffer, 100 μL of 10% Triton X-100, 12 μL of 10 mg/ml bovine serum albumin, 10 μL of 400 U/μL T4 DNA ligase) was added and samples were incubated at 16 °C for over 10 hours with rotation. Nuclei were pelleted by centrifugation for 5 min at 3,000Xg and were wash with 1xPBS. Pellets were then resuspended in 400ul 1xPBS, and add 16.7ul 20mg/ml proteinase K and 40ul 10%SDS, incubate at 55°C for 30min. The sample was added with 43.3ul 5M Nacl, 12ul 0.5M EDTA, 24ul 1M DTT, 50ul 10%SDS, and Incubate at 68°C overnight. Cool sample at RT, and add 1250ul pure ethanol and 50ul 3M sodium acetate, pH 5.2. Mix by inverting and incubate at -80°C for over 1h. Centrifuge at 13,000 Xg, 4°C for 20 minutes. Keep the tubes on ice after spinning and carefully remove the supernatant by pipetting. Wash with 800μl of 70% ethanol twice and centrifuge at 13,000g for 5 minutes. Dissolve the pellet in 100μl of 1X Tris buffer (10 mM Tris-HCl, pH 8) and incubate at 37°C for 15 minutes to fully dissolve the DNA.

Quantify DNA by Qubit dsDNA High Sensitivity Assay (Life Technologies, Q32854). To make the biotinylated DNA suitable for high-throughput sequencing using Illumina sequencers, shear to a size of 300-500bp. Transfer sheared DNA to a fresh 1.5ml tube, and add equal volume of 2X Binding Buffer (2X BB: 10mM Tris-HCl (pH 7.5); 1mM EDTA; 2M NaCl), then centrifuge at 13,000g for 5 minutes. Prepare for biotin pull-down by washing 50μl of 10mg/ml Dynabeads MyOne Streptavidin C1 beads (Life technologies, 65002) with 400μl of 1X Tween Washing Buffer (1X TWB: 5mM Tris-HCl (pH 7.5); 0.5mM EDTA; 1M NaCl; 0.05% Tween 20). Separate on a magnet and discard the solution. Resuspend the beads with sheared DNA supernatant. Incubate at room temperature for 30 minutes with rotation to bind biotinylated DNA to the streptavidin beads. Separate on a magnet and discard the solution. Wash the beads by adding 500μl of 1X TWB. Heat the tubes on a Thermomixer at 55°C for 2 min with mixing. Reclaim the beads using a magnet and discard supernatant. Repeat wash twice. Resuspend beads in 100ul 1X Tris buffer (10 mM Tris-HCl, pH 8) and transfer the beads to a new PCR tube. Reclaim beads and discard the buffer. Resuspend beads in 50ul 1X Tris buffer (10 mM Tris-HCl, pH 8). Then the the beads binding with fragmented DNA was treated with the End Repair/dA-Tailing Module (NEB, E7546L) and Ligation Module (NEB, E7595L) following the operation manual. After ligation, the beads were washed with 1X TWB twice and 1X Tris buffer (10 mM Tris-HCl, pH 8). Beads were resuspended in 20 μL of 1X Tris buffer (10 mM Tris-HCl, pH 8). The Hi-C library was amplified for 10 cycles of PCR with Q5 master mix (NEB, M0492L) following the operation manual. PCR products were confirmed by analyzing 1 μL of product using the FlashGel System (Lonza, 57063). PCR was continued with additional cycles until bright DNA bands were seen. A bottle of Agencourt AMPure XP beads (Beckman Coulter, A63881) was warmed to room temperature and gently shaken to resuspend the magnetic beads. 100 μL of beads was added to 200 μL of diluted PCR product (0.5X volumes). Samples

were mixed by pipetting and incubated at room temperature for 10 min. Beads were pelleted on a magnet and the clear solution was transferred to a new tube. Another 30 µL of beads was added to the clear solution (0.65X volume), mixed by pipetting, and incubated at room temperature for 10 min. Keeping the beads on the magnet, samples were washed twice with 200 µL of 70% ethanol (freshly made) without mixing. Ethanol was then completely removed. Beads were left on the magnet for 5 min to allow the remaining ethanol to evaporate. DNA was eluted by adding 20 µL of ddH2O, mixing by pipetting, and incubating at room temperature for 5 min. After separating on a magnet, the solution was transferred to a new tube. DNA was then quantified and sequenced using an Illumina sequencing platform.

RNA extraction and library preparation

Total RNA was isolated using Quick-RNA MicroPrep Kit (Zymo Research, R1050) according to the manufacturer's instructions. Then polyA+ RNA was purified from total RNA by using oligo-dT attached magnetic beads (NEB, E7490), then the purified RNAs were used to prepare RNA-seq library by NEBNext Ultra II Directional RNA Library Prep Kit for Illumina (NEB, E7765) according to the manufacturer's instructions. Libraries were pooled and sequenced in PE150 mode on the Illumina sequencing platform.

ChIP-seq library preparation

To depict the CTCF landscape and H3K27ac modification landscape in different developing brain regions, we performed ChIP-seq for the CTCF protein and H3K27ac modifications. Millions of cells were used for each ChIP-seq library construction. Typically, 10–40 million cells were used for a single ChIP experiment. Bulk tissue washed one time by PBS, then centrifuge on 4 ℃ with speed 800Xg. To crosslink protein–DNA complexes, containing 1% formaldehyde was added for 10 min at room temperature (RT). Crosslinking was quenched by adding 125 mM glycine for 5 min at RT. Cells were then rinsed

three times in ice-cold PBS containing complete protease inhibitor cocktail tablets (Sigma) and collected by scraping. Cells were pelleted and either stored at −80 °C until use or processed immediately. Cell pellets were lysed by buffer 1 (50 mMTris-HCl, pH 8.0, 10 mM EDTA, pH 8.0, 0.2% SDS, 1mM PMSF, 20mM Na-butyrate and cocktail proteinase inhibitor) for 40 min at 4 °C. Nuclei were then pelleted by centrifugation at 1000g. for 10 min at 4 °C. Sonication was carried out using a BioruptorTM UCD-400 TO set at a HIGH setting of 18 cycles, (30s on/30s off) which resulted in genomic DNA fragments with sizes ranging from 200 bp to 2 kb. Insoluble materials were removed by centrifugation at the max speed. for 10 min at 4 °C. The supernatant was transferred to a new tube. 10% of the ChIP sample (10 μL from 100 μL lysate) was saved as input material. The remaining lysate was incubated with antibodies for immunoprecipitation. The antibody incubation was carried out overnight at 4 °C.The beads bound by immune complexes were pelleted and washed three times with each of the following buffers: low-salt buffer (0.1% SDS, 1% Triton X-100, 2 mM EDTA, 20 mM Tris-HCl, pH 8.1, and 250 mM NaCl), high-salt buffer (0.1% SDS, 1% Triton X-100, 2 mM EDTA, 20 mM Tris-HCl, pH 8.1. In each wash, the beads were incubated with wash buffer for 5 min at 4 °C while nutating. The washed beads were then rinsed once with 1× TE buffer (10 mM Tris-HCl, pH 8.0, and 1 mM EDTA). Then, 10 μL Proteinase K was added to each sample, and samples were incubated at 65 °C for 8 h. The immunoprecipitated genomic DNA fragments were then extracted by Beckman XP beads and then back extracted with water. Libraries were sequenced in PE150 mode on the Illumina sequencing platform. Antibodies: CTCF (A1133, Abclonal) and H3K27ac (ab4729, Abcam).

Generation and Immunostaining of human cortical organoids

Human H9 embryonic stem cells were maintained in Essential 8 Medium (A1517001, Gibco) on 6-well plates coated with Matrigel (354277, Corning). On

day 0, we dissociated the target cell colonies into single cells by Accutase (A1110501, Gibco) and suspended them as 100 cells/μL in KSR medium containing DMEM/F-12 (11320082, Gibco), 20% KSR (A3181502, Gibco), 2mM GlutaMax-I (35050061, Gibco), 0.1mM NEAA (11140076, Gibco), 0.1mM beta-mercaptoethanol (21985023, Gibco), 10μM SB431542(1614, TOCRIS), 0.1μM LDN-193189 (6032, TOCRIS), 3μM endo-IWR1 (3532,TOCRIS). Then we transferred the cells to 96-well V-bottom plates. We replaced half of the medium every day until day 18. On day 18, the medium was replaced with neural induction medium containing DMEM/F12, 1:100 N2 supplement (17502048, Gibco), 2 mM GlutaMax-I, 0.1 mM NEAA, 0.1 μM beta-mercaptoethanol and organoids were transferred to 24-well low-cell-adhesion plate. Half of the media was replaced on alternate days.

Organoids were fixed with 4% paraformaldehyde in PBS for 1h at 4°C, cryoprotected in 30% sucrose and embedded in optimal cutting temperature medium. 25 μm cryosections were collected on superfrost slides using a Leica CM3050S cryostat. The slices were blocked with 10% donkey serum in PBS with 0.1% Triton X-100, and then incubated with primary antibodies: PAX6 (901301, BioLegend, 1:200 dilution), TUBB3 (801201, BioLegend, 1:200 dilution). Binding was visualized using an appropriate Alexa Fluor 594 or Alexa Fluor 647 conjugated donkey anti-mouse. Images were collected using an Olympus FV3000 confocal microscope.

Knock-out of *SLN* gene enhancer

We designed sgRNA to target the putative sequences of *SLN* enhancers using the GPP sgRNA Designer (CRISPRko) (https://portals.broadinstitute.org), then optimized sgRNA design to maximize activity and minimize off-target effects of CRISPR-Cas9. The sgRNA sequences were as follow: SLN sgRNA1 AAG ACC ATT TAA AAC TAC GG, sgRNA2 TAG CTA AGT TAG TCC AGC AA. The knock-out sequences were shown in Source Data 1. The sgRNA were

cloned into the HP180-CBH-Cas9-CMV-EGFP or HP180-CBH-Cas9-CMV-RFP plasmids. Electroporation was performed using Lonza AMAXA 4D-Nucleofector and the Human H9 cells expressing the plasmids were cultured in two days. Then the GFP/RFP double positive cells were detected and isolated into a 96 well plate coated with Matrigel by FACS (one cell each well). Genomic DNA amplification of the target sequence was conducted to confirm the knock-out of enhancer using the following primers: SLN forward ACC TGG TAA TAC AGG TGG ACC AT, reverse TGG GAA GGT TCA GAC TCT TGG AAC C.

qRT-PCR of target genes

Total RNA samples were isolated from enhancer-knockout cells using SV Total RNA Isolation System (Z3100, Roche). RNA concentration was measured with a Nanodrop and RNA integrity were validated by agarose gel electrophoresis. cDNA was prepared using PrimeScriptII 1st Strand cDNA Synthesis Kit (6210, TaKaRa). qRT-PCR was proceeded on a PCR biosystems QuantStudio 7 Flex instrument (Applied Biosystems) with FS Universal SYBR Green Master (4913914001, Roche). We used the following qRT-PCR primers: SLN forward ATG GTC CTG GGA TTG ACT GAG, reverse GTG CCC TCG GAT GGA GAA TG. GAPDH (Endogenous Control) forward CCA TGG GTG GAA TCA TAT TGG A, reverse TCA ACG GA TTT GGT CGT ATT GG. The expression level of target genes was normalized to GAPDH and analyzed using ΔΔCT. The experiment was repeated three times independently with the similar results.

Hi-C data processing

The HiCExplorer suite[59-61] was used for the processing of Hi-C data. Reads were first mapped to human genome hg19 using bwa mem with parameters '-E50 -L0'. Then, all read pairs which were not uniquely mapped (mapping score <15), dangling end reads, same fragment reads, self-circled reads, self-ligation reads and other invalid Hi-C reads were discarded. Details about Hi-C data

quality were summarized in Supplementary Table S1. After removing duplication, reads were used to generate raw Hi-C matrix using ‘hicBuildMatrix’. In the raw contact matrix, rows and columns with zero or small total counts were removed because these bins were mostly from repetitive regions. After filtering low count bins, the matrices were corrected by the ICE method[62] using ‘hicCorrectMatrix’. The reproducibility for Hi-C data was measured by GenomeDISCO[63] reproducibility score. We calculated GenomeDISCO reproducibility score at 200kb resolution for Hi-C replicates.

RNA-seq data processing

We used Refseq gene annotation from the UCSC genome browser. Low quality RNA-seq reads and adaptor sequences were removed by Trimmomatic v0.32. Then reads were mapped to the hg19 genome by STAR[64] with default parameters. Gene expression FPKM was calculated by HOMER software. RNA-seq tracks for visualization were generated by bamCoverage program in Deeptools2 with parameter ‘–normalizeUsingRPKM’.

ChIP-seq data processing and peak calling

For ChIP-seq data, the low quality reads were removed by Trimmomatic v0.32 and then were mapped to human genome hg19 by bwa mem. Reads with low mapping quality (MAPQ < 10) were filtered and PCR duplicated reads were removed by Picard tools. The peaks were called by MACS2 with parameters “-g hs -B -p 1e-5 -f BAMPE --SPMR” for narrow peak calling. The tracks of ChIP-seq data (processed with FPKM) were visualized on the WashU Epigenome Browser and IGV Browser. The published fetal brain histone modification and DNase tracks[65] and ATAC peaks[66,67] were downloaded from Roadmap data resource and GEO database.

Insulation scores

Insulation score was calculated as described previously[68] with the public

code on Github (*matrix2insulation.pl*; https://github.com/dekkerlab/giorgetti-nature-2016). A sliding 480 kb X 480 kb square along the matrix diagonal was used. The IQR mean signal within the square was then assigned to each 40 kb diagonal bin. This procedure was then repeated for all 480 kb diagonal bins. The insulation score was normalized relative to all of the insulation scores across each chromosome by calculating the log2 ratio of each bin's insulation score versus the mean of all insulation scores. Valleys/minima along the normalized insulation score vector represent the loci of reduced Hi-C interactions that occur across the bin. These valleys/minima are interpreted as areas of high local insulation.

A/B compartments, TADs and Chromatin Loops

We used HOMER[69] software with parameters '–res 100,000, -superRes 400,000' to obtain the PC1, PC2 and PC3 value. Because sometimes PC2 or PC3 value reflect A/B compartments, we manually checked of PC1, PC2 and PC3 track with gene density and the plaid pattern in the correlation heatmaps along each chromosome and got final 'PC1' list. The direction of the Eigen (PC) values is arbitrary, and therefore positive values were set to 'A' and negative values were set to 'B' based on their association with gene density. The spearman's correlation between the A/B compartment PC1 value and DNase chromatin accessibility signal/histone modification signal enrichment were calculated at 100kb bin resolution. We identified A/B compartment dynamics regions as statistically significant variability in PC1 values across developmental stages using ANOVA with adjusted p value < 0.05.

TADs and TAD boundaries were identified based on public TAD separation score method[59-61] with 'hicFindTADs' command in HiCExplorer suite. The TAD-separation scores in human embryos were calculated at 40kb resolution using parameters "--minDepth 300,000 –maxDepth 3,000,000 --step 300,000 --minBoundaryDistance 400,000 –thresholdComparisons 0.01 –

correctForMultipleTesting fdr –delta 0.01”. The regions with q < 0.01 were reported as boundaries. TADs were identified as regions between two boundaries. Differential Domains were identified by the ‘Diffdomain’ software.

We applied ‘hicDetectLoops’ [59-61] to detect enriched/significant interaction peaks within chromosomes at 10 kb resolution. Briefly, for each genomic distance, a negative binomial distribution is computed and only interaction bin pairs with p.value <0.05 are accepted as candidate loops. Next, each candidate is compared to its neighborhood. This neighborhood is defined by the window size in the x and y dimension around the candidate. Per neighborhood only one candidate is considered, therefore only the candidate with the highest peak values is accepted. We performed a parameter sweep for the chromatin loops and merged all loops into the final loop annotation list.

For a given gene such as *SLN* or a brain disease-associated SNP, we also applied the method introduced in Won et al., 2016[70] to identify significant Hi-C interactions. Briefly, to avoid significant Hi-C interactions affecting the distribution fitting as well as parameter estimation, the lowest 95 percentiles of Hi-C contacts were used and zero contact values were also removed. Using these background Hi-C interaction profiles, the distribution of Hi-C interactions at each distance for each chromosome using fitdistrplus package. The weibull distribution was used to calculate the statistical significance for a given Hi-C interaction at the matched chromosome and distance. Hi-C contacts with p.value <0.0001 were selected as significant interactions. The interaction profile for a given locus was visualized by Gviz package.

<u>Feature-free chromatin structure difference detection for different brain regions</u>

To find out the dynamics of chromomatin 3D structures in different human brain regions, we employed a feature-free method CHESS[71] to detect differences. CHESS can robustly identify and classify specific similarities or differences and features in chromatin contact data like Hi-C data. CHESS

applies the structural similarity index (SSIM) which was widely used in image analysis to chromatin contact matrices, assigning a structural similarity score and an associated P value to pairs of genomic regions. Briefly, to calculate the similarity between a reference (R) and query (Q) matrix, their entries were first divided by the expected contact intensity at the respective distance to remove the distance dependency of pairwise contact probabilities characteristic of Hi-C matrices. CHESS then scaled the matrices to equal size and calculates the SSIM between R and Q. The SSIM score (S) was a single value, combining brightness, contrast and structure differences between two matrices. Brightness was calculated as the mean of the signal intensity. Contrast was calculated as the variance in signal. The structure term was calculated as the correlation between signal values of two matrices. S was then defined as a weighted product of these three components, which were scaled such that S ranged between −1 (inversion) and 1 (identity), where S = 0 indicates no similarity. S can be used directly to quantify changes in chromatin contacts within windows of a given size across the genome: for identical matrices S = 1 and the lower the score the larger the change. CHESS version 0.3.7 was run using window sizes of 2 Mb, with a 500 kb step size, for data binned at 25 kb resolution, and 4 Mb windows with a 1 Mb step size for data binned at 50 kb resolution.

The dynamic gene expression clusters during the brain development

To find out how the time-course gene expression dynamics occur during the multiple human brain region development, we filter out the unexpressed genes (FPKM<0.5) in all stages and use 'TCseq' package to capture the temporal patterns of the transcriptome by soft clustering. The genes with membership > 0.5 for each cluster were used for GO enrichment analysis by the 'clusterProfiler' package.

Super enhancers

All H3K27ac ChIP-seq data sets were aligned using BWA (version 0.7.15) mem to build version hg19 of the human genome. We randomly downsampled 35 million reads from treat ant control group in each sample, then were used to call peak by MACS2. We used ROSE software[72] to identify super-enhancers. In order to accurately capture dense clusters of enhancers, we allowed regions within 12.5 kb of one another to be stitched together and excluded regions contained within +/- 2500kb from TSS in order to account for promoter biases. Only if the number of constituents stitched more than 2, the super enhancers were considered to be valid and were used to further processing.

GWAS SNP analysis and identification of target genes

GWAS SNPs for a total of 8 neuropsychiatric disorders or mental trait (ASD, SCZ, ADHD, MDD, AD, PD ALS and IQ) were mined from the GWAS Catalog[73] (September 2019) using a P value threshold of 10^{-6} (details in Supplementary Table S5). The GWAS SNPs were expanded to sets of linked SNPs using HaploReg 4.1(Ward and Kellis, 2012) at an LD threshold of 0.8 according to the reported study population(s) for each SNP. All SNPs were fitted to hg19 and filtered for duplicates by position. If genes have significant interactions/loops with the GWAS SNPs in a certain human brain region, we identified the genes as GWAS SNP-linked genes.

Statistical analysis

R and Prism were used for statistics analysis. Wilcoxon rank sum test was used for statistical significance (Fig. 3C; Supplementary Fig. S2C) with the wilcox.test function in R. Two-sided unpaired t.test was used for statistical significance (Fig. 4f, h; Supplementary Fig. S10g). The GO functional enrichment was calculated by the ‘clusterProfiler’ package and the DAVID. For violin plots, the white dots represent the median values, boxes and whiskers

represent the 25th/50th/75th percentiles and 1.5X the interquartile range, respectively. For significance marks, * means p.value < 0.05, ** means p.value < 0.01, *** means p.value < 0.001, **** means p.value < 0.0001 and n.s. represents no significance (p.value > 0.05). All of the statistical details can be found in the figure legend and methods.

Code availability

All analyses in our study were performed with open-source packages as described in the “Materials and methods” section. All relevant codes are available from the corresponding authors upon reasonable request.

Supplementary Tables

Table S1. Hi-C data summary in different developing brain regions (separate file)

Table S2. GO terms for genes with AB compartment dynamics in different brain regions (separate file)

Table S3. Chromatin loops in different developing brain regions (separate file)

Table S4. GWAS SNP summary for neuropsychiatric disorders and traits (separate file)

Supplementary Data Source 1. SLN enhancer knock-out sequence by Sanger Sequencing

References

58 Rao, S. S. *et al.* A 3D map of the human genome at kilobase resolution reveals principles of chromatin looping. *Cell* **159**, 1665-1680, doi:10.1016/j.cell.2014.11.021 (2014).

59 Ramirez, F. *et al.* High-resolution TADs reveal DNA sequences underlying genome organization in flies. *Nature communications* **9**, 189, doi:10.1038/s41467-017-02525-w (2018).

60 Wolff, J. *et al.* Galaxy HiCExplorer 3: a web server for reproducible Hi-C, capture Hi-C and single-cell Hi-C data analysis, quality control and visualization. *Nucleic Acids Res* **48**, W177-W184, doi:10.1093/nar/gkaa220 (2020).

61 Wolff, J. *et al.* Galaxy HiCExplorer: a web server for reproducible Hi-C data analysis, quality control and visualization. *Nucleic Acids Res* **46**, W11-W16, doi:10.1093/nar/gky504 (2018).

62 Imakaev, M. *et al.* Iterative correction of Hi-C data reveals hallmarks of chromosome organization. *Nat Methods* **9**, 999-1003, doi:10.1038/nmeth.2148 (2012).

63 Ursu, O. *et al.* GenomeDISCO: a concordance score for chromosome conformation capture experiments using random walks on contact map graphs. *Bioinformatics* **34**, 2701-2707, doi:10.1093/bioinformatics/bty164 (2018).

64 Dobin, A. *et al.* STAR: ultrafast universal RNA-seq aligner. *Bioinformatics* **29**, 15-21, doi:10.1093/bioinformatics/bts635 (2013).

65 Roadmap Epigenomics, C. *et al.* Integrative analysis of 111 reference human epigenomes. *Nature* **518**, 317-330, doi:10.1038/nature14248 (2015).

66 de la Torre-Ubieta, L. *et al.* The Dynamic Landscape of Open Chromatin during Human Cortical Neurogenesis. *Cell* **172**, 289-304 e218, doi:10.1016/j.cell.2017.12.014 (2018).

67 Markenscoff-Papadimitriou, E. *et al.* A Chromatin Accessibility Atlas of the Developing Human Telencephalon. *Cell* **182**, 754-769 e718, doi:10.1016/j.cell.2020.06.002 (2020).

68 Giorgetti, L. *et al.* Structural organization of the inactive X chromosome in the mouse. *Nature* **535**, 575-579, doi:10.1038/nature18589 (2016).

69 Heinz, S. *et al.* Simple combinations of lineage-determining transcription factors prime cis-regulatory elements required for macrophage and B cell identities. *Mol Cell* **38**, 576-589, doi:10.1016/j.molcel.2010.05.004 (2010).

70 Won, H. *et al.* Chromosome conformation elucidates regulatory relationships in developing human brain. *Nature* **538**, 523-527, doi:10.1038/nature19847 (2016).

71 Galan, S. *et al.* CHESS enables quantitative comparison of chromatin contact data and automatic feature extraction. *Nature genetics* **52**, 1247-+, doi:10.1038/s41588-020-00712-y (2020).

72 Whyte, W. A. *et al.* Master transcription factors and mediator establish super-enhancers at key cell identity genes. *Cell* **153**, 307-319, doi:10.1016/j.cell.2013.03.035 (2013).

73 Xie, Y. H. *et al.* Cloning and characterization of human IC53-2, a novel CDK5 activator binding protein. *Cell Res* **13**, 83-91, doi:10.1038/sj.cr.7290153 (2003).
