# Supplementary material for: Spatiotemporal 3D chromatin organization across multiple brain regions during human fetal development
Source: Cell Discov. 2025 May 16;11:50. doi: 10.1038/s41421-025-00798-w (PMC12081887; doi:10.1038/s41421-025-00798-w)
Supplement: Supplementary file 2 — Supplementary Data Source 1 [file 41421_2025_798_MOESM2_ESM.pdf]

The Knock-out Sequence of SLN Enhancer

```
Control:                            GTAATACAGGTGGACCATGATCTCATATTA
SLN Enhancer knock-out sequence:    GTAATACAGGTGGACCATGATCTCATATTA

GCAAATGTTTTTCATACCTATCATTATTATTTGGGGCTTGTGAGTTTCTGACTTATTTCT
GCAAATGTTTTTCATACCTATCATTATTATTTGGGGCTTGTGAGTTTCTGACTTATTTCT

TATGTTATCCCTCATATTACACATACCTCCGTAGTTTTAAATGGTCTTTCAGGCCAATGA
TATGTTATCCCTCATATTACACATACCTCCG-----------------------------

ATATAAATAAATATTATGAGATGAGGTGAGGTGGTTAGCAGTGACTTCACATTAATTGG
------------------------------------------------------------

TTTTAATCAAACCCAAGGAAATTGGGGTGAGGTGAGGTGAGGTGGTTAGACAAGCCT
------------------------------------------------------------

TGGCAAAGAATTCAGGAAACAAAAAATTAGGGACAAATTGTTAAAGACAAAAATCTAG
------------------------------------------------------------

GACCCAAAGAAAATACAAATTCTTTTCAGTGTATCAGGCACCTTTGACCAGGAGGTG
------------------------------------------------------------

GCAGCAGTGGAAGCAAGAACGTCTAGAAACAAAGTATCCTGGGGCAAATACCAACAC
------------------------------------------------------------

TATTGAAACCACTTTTGCAAAATTATAACTGAGGAAATGATGACAGTGAAAGAAATCAG
------------------------------------------------------------

ATCTAACTGTCTCTGTCTTGCTTCTAACCTTTAAGCTGTCCTTGTTCATTCCTGGGCA
------------------------------------------------------------

CAGGTCAAACTAACTTTGAGAAGGAATTCAGTTCATGGTTTGACTCTGAAACAAAATT
------------------------------------------------------------

GATAACAGCCCTTTCCCAAAAAGACCCCCTTCTTGCCTGGGGACCAGTCTGCCTTTG
------------------------------------------------------------

CTGGACTAACTTAGCTACAAGATTGAAAATTACAGTTTAGGGGTCATACAATCTCTGGT
CTGGACTAACTTAGCTACAAGATTGAAAATTACAGTTTAGGGGTCATACAATCTCTGGT

TCCAAGAGTCTGAACCTTCCCAAATTGCTCCTGGGGATAACAT
TCCAAGAGTCTGAACCTTCCCAAATTGCTCCTGGGGATAACAT
```
